# MFN2 alleviates mitochondrial calcium overload in sepsis-induced myopathy by modulating GRP75–VDAC1 interaction

**DOI:** 10.1186/s13395-026-00441-6

**Published:** 2026-08-13

**Authors:** Xuexin Li, Fei He, Yun Wang, Bowen Sun, Jie Li, Yuchen Tang, Li Liu

**Affiliations:** 1 Department of Anesthesiology, The Fourth Affiliated Hospital, Southwest Medical University, No. 168, Tianfu New Area, Meishan, Sichuan 620000 China; 2 Anesthesiology and Critical Care Medicine Key Laboratory of Luzhou, Southwest Medical University, Luzhou, Sichuan Province 646000 China

**Keywords:** MFN2, MAM, Mitochondrial calcium overload, IP3R–GRP75–VDAC1 complex, Sepsis, Skeletal muscle atrophy

## Abstract

**Background:**

Sepsis-induced myopathy (SIM) is characterized by skeletal muscle dysfunction associated with mitochondrial calcium overload. The mitochondria-associated endoplasmic reticulum membrane (MAM) plays a crucial role in mediating mitochondrial calcium uptake. Although MFN2 and the IP3R–GRP75–VDAC1 complex have each been implicated in mitochondrial calcium regulation, their respective contributions and potential interplay in the pathogenesis of SIM remain poorly understood.

**Methods:**

A murine sepsis model was established by cecal ligation and puncture (CLP). Skeletal muscle function was assessed by compound muscle action potential (CMAP) recording and forelimb grip strength testing. Gastrocnemius muscle histology was evaluated by H&E and WGA staining. Tibialis anterior muscles were analyzed by immunofluorescence and transmission electron microscopy (TEM) to characterize MAM ultrastructure. Protein expression was quantified by western blotting, and MFN2 mRNA levels were measured by qPCR. Co-immunoprecipitation was performed to assess GRP75–VDAC1 protein interaction. Mitochondrial and cytoplasmic calcium levels were monitored using Rhod-2 AM and Fluo-4 AM, respectively.

**Results:**

Sepsis induced skeletal muscle atrophy and dysfunction both in vivo and in vitro. IP3R-dependent calcium transfer at MAM was associated with pathological mitochondrial calcium overload, and pharmacological IP3R inhibition with 2-APB attenuated mitochondrial calcium uptake and ameliorated muscle atrophy. Concurrently, sepsis-induced MFN2 downregulation was associated with reduced ER-mitochondria distance, further promoting mitochondrial calcium overload. AAV-mediated MFN2 overexpression restored ER-mitochondria distance and was associated with reduced VDAC1 protein expression, attenuated GRP75–VDAC1 interaction, collectively contributing to mitigation of mitochondrial calcium overload and amelioration of skeletal muscle atrophy.

**Conclusions:**

MFN2 attenuates sepsis-induced mitochondrial calcium overload through two complementary mechanisms: increasing ER-mitochondria distance and modulating GRP75–VDAC1 interaction and IP3R–GRP75–VDAC1 complex assembly. These findings identify MFN2 as a potential regulator of MAM-mediated mitochondrial calcium homeostasis in SIM and suggest it may represent a therapeutic target for sepsis-induced skeletal muscle dysfunction.

**Supplementary Information:**

The online version contains supplementary material available at https://doi.org/10.1186/s13395-026-00441-6.

## Introduction

Sepsis-induced myopathy (SIM) is a rapidly developing myopathy in patients with sepsis that is characterized by central nervous system or muscular abnormalities. It manifests primarily as muscle weakness, atrophy, and alterations in bioenergetics [1]. Studies have shown that patients with severe sepsis suffer more than 10% acute muscle atrophy in the lower extremities, alongside a 50% reduction in the strength of the ventilatory and locomotor muscles within two weeks of hospitalization [2, 3]. The pathophysiological mechanisms underlying SIM remain complex and unclear. However, mitochondrial structural and functional damage resulting from impaired mitochondrial calcium delivery has been identified as a potential early event in the SIM [4]. This dysfunction may represent a critical therapeutic target for the treatment of SIM.

The sarcoplasmic reticulum (SR) is found in skeletal muscle fibers and contains more than 80% of the calcium stored in resting fibers [5]. The region where mitochondria are structurally and functionally connected with the endoplasmic reticulum (ER)/SR is referred to as the mitochondria-associated ER/SR membrane (MAM) [6]. This structure accounts for approximately 5–20% of the surface area of the mitochondrial network and facilitates the efficient transfer of calcium from the ER to the mitochondria, thereby promoting oxidative metabolism [7]. Alterations in the ER-mitochondria interface, such as changes in organelle morphology and distance, can influence cellular responses and signaling pathways [8]. Under physiological conditions, the ER-mitochondria distance is typically 10–30 nm, which plays a crucial role in mitochondrial calcium uptake. A decrease in distance can lead to mitochondrial calcium overload and cellular damage [9, 10]. Recent studies have shown that MAM-mediated calcium transfer dysfunction is implicated in skeletal muscle damage, including insulin resistance [11], Duchenne muscular dystrophy [12], sarcopenia [13], and statin-induced muscle dysfunction [14]. However, there have been no reports on the role of MAM-mediated alterations in mitochondrial calcium uptake in SIM.

MFN2 is a GTPase enzyme located in the ER membrane and outer mitochondrial membrane (OMM) and was the first MAM tethering protein to be identified [15]. MFN2 forms homodimers that regulate ER-mitochondria interactions and has been extensively studied as a “distance holder” of the MAM [16]. However, the role of MFN2 in MAMs remains controversial. Some studies have reported that decreased MFN2 expression increases the ER-mitochondria distance, reducing mitochondrial calcium uptake [17]. Conversely, more recent research suggests that MFN2 acts as a protective tethering antagonist, preventing excessive and potentially toxic proximity between the two organelles [18]. Filadi et al. [19] demonstrated that the ablation of MFN2 increased close contact between the ER and mitochondria, thereby increasing IP3R-induced calcium release from the ER to the mitochondria, resulting in mitochondrial calcium overload and triggering cell death. Additionally, recent studies have highlighted the increasing importance of the IP3R–GRP75–VDAC1 complex in maintaining MAM integrity and regulating calcium signaling [20]. Previous study has demonstrated that IP3R–GRP75–VDAC1 complex is regulated by MAM-associated proteins [21]. However, the exact role of MFN2 in modulating this complex remains unclear and warrants further investigation.

Therefore, this study aims to investigate whether MFN2-mediated disruption of MAM calcium transfer contributes to the pathogenesis of SIM and to explore the potential interaction between MFN2 and the IP3R–GRP75–VDAC1 complex. These insights could provide a theoretical foundation for the development of clinical strategies targeting SIM.

## Materials and methods

### Reagents and antibodies

2-Aminoethyl diphenylborinate (2-APB) was purchased from Selleck Chemicals (S6657, Shanghai, China). Histamine was purchased from MedChemExpress (HY-B1204, Shanghai, China). Pluronic F-127 was purchased from Solarbio (P6790, Shanghai, China). Sera, including fetal bovine serum (FBS), horse serum (HS), and Dulbecco’s modified Eagle’s medium (DMEM), were purchased from Cegrogen Biotech Gmbh (Eupen, Germany). The fluorescent calcium indicators, including Rhod-2 AM (R1244) and Fluo-4 AM (F14217), were obtained from Invitrogen (California, USA). TRIzol, HiScript III RT SuperMix for Qpcr (+ gDNA wiper) and ChamQ Universal SYBR Qpcr Master Mix were obtained from Vazyme (Nanjing, China).

MuRF1, Atrogin-1, MFN2 and GAPDH antibodies were purchased from Santa Cruz Biotechnology (Santa Cruz, CA). IP3R, VDAC1, LC3B and p62 antibodies were purchased from Abcam (Cambridge, MA). MCU antibody and secondary antibodies for immunofluorescence staining were purchased from Cell Signaling Technology (Beverly, MA, USA). GRP75, PINK1, and Parkin were purchased from Proteintech (Wuhan, China). All secondary antibodies for western blotting were purchased from Beyotime (Shanghai, China). Detailed information on the primary antibodies and their dilutions for western blotting is described in Supplementary Table 1, and the information for immunofluorescence staining is described in Supplementary Table 2.

### Animals and groups

Male C57BL/6J mice (eight weeks old) from Huafukang Biotechnology Co., Ltd. (China) were housed in a 12 h light/12 h dark environment with free access to food and water. After one week of adaptation, the mice were randomly divided into four groups: 24- and 96-h sham-operated groups (sham-24 h, sham-96 h, *n* = 12/group) and cecal ligation and puncture (CLP) groups (CLP-24 h, CLP-96 h, *n* = 20/group).

For in vivo gene delivery, adeno-associated virus serotype 2/9 (AAV2/9) vectors were obtained from Hanbio Biotechnology (HH20240511CDNWH-AAV01, Shanghai, China). The AAV vectors contained either a negative control sequence (AAV-CON) or a mouse MFN2 coding sequence (AAV-MFN2), both driven by a cytomegalovirus promoter and packaged within the AAV9 capsid with AAV2 inverted terminal repeats. Intramuscular injections were performed using a micro syringe (Hamilton, USA) at a viral titer of [2.2 × 10^12^ vg/mL]. A total volume of 24 µL was injected into the tibialis anterior (TA) muscle and 30 µL into the gastrocnemius (GAS) muscle per animal, with each muscle receiving injections at three different sites to ensure even distribution. In each animal, the right TA and GAS muscles were injected with AAV-CON, and the contralateral left muscles were injected with AAV-MFN2. Mice were allowed to recover for four weeks post-injection to ensure adequate transgene expression prior to further analysis, and successful MFN2 overexpression was subsequently confirmed at both the mRNA and protein levels by qPCR and western blotting, respectively. All animal care and experimental procedures were approved by the Animal Ethics Committee of Southwest Medical University (SWMU20240052).

### Model of sepsis

The sepsis model was established via CLP as described previously [22, 23]. Briefly, the mice were anesthetized with sodium pentobarbital (50 mg/kg) after 12 h of fasting without water, after which a midline incision in the abdomen was made to expose the distal 1/2 of the cecum. The cecum was ligated, and a 21-gauge needle was used to puncture the central segment of the cecum. A small amount of cecal contents was squeezed through the puncture wound, the cecum was returned to the abdominal cavity, and the surgical incision was sutured layer by layer. In the sham group, only cecal exploration was performed. Postoperative resuscitation was performed with subcutaneous injections of normal saline (50 mL/kg) and buprenorphine (0.05 mg/kg) for analgesia. Mice were also administered imipenem (20 mg/kg) intraperitoneally immediately after surgery and every 24 h thereafter.

### Animal serum inflammatory factor measurements

After CLP modeling, five mice from each group were randomly selected. Euthanasia was performed by intraperitoneal injection of sodium pentobarbital (100 mg/kg). Blood samples were immediately collected by cardiac puncture and centrifuged at 1000 × g for 15 min at 4 °C. Serum concentrations of IL-6, TNF-α, and IL-1β were measured using ELISA kits (MEIMIAN, China) according to the manufacturer’s instructions.

### Compound muscle action potential of the sciatic nerve

The compound muscle action potential (CMAP) assay represents a well-established and sensitive approach for assessing neuromuscular function in mice [24]. CMAP was recorded by placing a stimulating electrode subcutaneously on each side of the sciatic notch, with an inter-electrode distance of approximately 2 cm. The active recorder was positioned at the GAS muscle, and the reference recorder was located at the paw of the hind limb of mouse. The amplitude and latency of the CMAP were tested as the primary parameters.

### Forelimb grip strength test

Forelimb grip strength was assessed using a grip strength meter (ZS-ZL; Zhongshi Dichuang, Beijing, China) as previously described [25]. Briefly, each mouse was gently pulled by the tail at a constant speed for 2–3 s, during which it instinctively grasped a horizontal metal bar connected to a force transducer. The peak traction force was automatically recorded and displayed on a digital screen. Three consecutive measurements were obtained per mouse and averaged for subsequent analysis. To account for differences in body mass, grip strength values were normalized to body weight, with lower ratios indicative of impaired skeletal muscle strength [26].

### Muscle weight measurement

Muscle mass loss was evaluated by measuring the wet weights of the TA and GAS muscles. At 24 h and 96 h following sham surgery or CLP, muscles from each experimental group were carefully dissected, weighed using an analytical balance, snap-frozen in liquid nitrogen, and stored at -80 °C until further analysis.

### H&E staining and WGA staining

Mouse skeletal muscle tissues were fixed in 4% paraformaldehyde and subsequently embedded in paraffin. Transverse Sect.  (6 μm thick) of the GAS muscle were cut and processed for histological analysis. For H&E staining, sections were stained with hematoxylin and eosin according to standard protocols. For wheat germ agglutinin (WGA) staining, sections were incubated with iF555-conjugated WGA to delineate the boundaries of individual muscle fibers and facilitate cross-sectional area quantification. Nuclei were counterstained with DAPI in PBST for 5 min at room temperature. The sections were examined and imaged using a fluorescence microscope (Leica, Tokyo, Japan).

### Immunofluorescence staining

Paraffin-embedded sections of the TA muscle were deparaffinized in xylene for 30 min and rehydrated through a graded ethanol series for 15 min, followed by heat-mediated antigen retrieval. Non-specific binding was blocked by incubating sections with 5% BSA in PBS for 1 h at room temperature. Sections were subsequently incubated with the primary antibodies overnight at 4 °C, followed by incubation with the corresponding secondary antibodies for 1 h. Nuclei were counterstained with DAPI for 10 min. Immunofluorescence images were acquired using a confocal laser scanning microscope (OLYMPUS, Tokyo, Japan).

### Transmission electron microscopy

Freshly dissected TA muscle samples were fixed overnight in 2.5% glutaraldehyde at 4 °C, post-fixed in 1% osmium tetroxide, and subsequently embedded in Epon resin according to standard protocols. Ultrathin sections were contrasted sequentially with 1% uranyl acetate and lead citrate to enhance membrane contrast. For each sample, five representative fields containing transversely oriented muscle fibers were randomly selected and imaged using a JEOL JEM-2100 F transmission electron microscope (TEM; JEOL, Tokyo, Japan). Quantitative morphometric analyses were performed using ImageJ software (NIH, Bethesda, MD, USA). The MAM interface percentage was calculated by measuring the length of ER membrane in apposition to the OMM relative to the total mitochondrial perimeter. To quantify the ER-mitochondria distance, a minimum of three mitochondria per image were analyzed across 30–40 images per experimental group, and only ER membranes positioned within 10–30 nm of the OMM were included in the analysis. Given the relatively uniform thickness of individual MAMs, the inter-membrane distance was defined as the mean of three measurements taken at randomly selected positions between the opposing ER and mitochondrial membranes [10].

### Cell culture and in vitro inflammation models

The murine C2C12 myoblast cell line was maintained in DMEM supplemented with 10% fetal bovine serum at 37 °C in a humidified atmosphere containing 5% CO_2_. Upon reaching 80% confluence, differentiation was induced by replacing the growth medium with DMEM supplemented with 2% horse serum. Fully differentiated myotubes were harvested six days post-induction for subsequent experiments.

To establish an in vitro model of cellular inflammation, differentiated C2C12 myotubes were exposed to 2% (v/v) serum collected from sham-24 h or CLP-24 h mouse, and the control and experimental groups were labeled the SMS and CMS groups, respectively. To pharmacologically suppress IP3R-mediated calcium release, 2-APB was dissolved in DMSO and administered to cells at a final concentration of 50 µM for 1 h following 96 h of mouse serum treatment. An equivalent volume of DMSO served as vehicle control.

Myotube diameter was quantified as previously described by Ono et al. [27]. Briefly, the widths of 90–120 myotubes were measured at three evenly spaced points along their longitudinal axis from at least 15 randomly selected fields per condition using ImageJ software, and the three measurements were averaged to yield the diameter of each individual myotube. Results are reported as the mean myotube width per experimental group.

### Stable lentiviral transduction of C2C12 myoblasts

To generate stable MFN2-overexpressing C2C12 myoblast cell lines, cells were seeded at a density of 2 × 10^3^ cells/cm^2^ and transduced with MFN2-expressing lentiviral particles in the presence of 5 µg/mL polybrene to enhance transduction efficiency. After 24 h, the lentivirus-containing medium was removed and replaced with fresh complete culture medium supplemented with 2 µg/mL puromycin. Cells transduced with an empty lentiviral vector were used as the negative control and designated as the LVs-CON group. Following 5 days of puromycin selection, stably transduced cells overexpressing MFN2 (LVs-MFN2 group) were validated by western blotting and qPCR prior to subsequent experiments.

### Dynamic in vitro calcium measurements

To monitor ER-to-mitochondria calcium transfer dynamics, C2C12 cells were seeded and differentiated in 96-well plates at a density of 1 × 10^5^ cells/well and subjected to the indicated pharmacological treatments. Mitochondrial calcium was measured using the cell-permeant fluorescent indicator Rhod-2 AM (2 µM; excitation: 552 nm, emission: 581 nm), and cytosolic calcium was monitored using Fluo-4 AM (2 µM; excitation: 488 nm, emission: 506 nm). Both probes were prepared in loading buffer containing 0.02% Pluronic F-127 to facilitate cellular uptake. C2C12 myotubes were incubated with the respective fluorescent indicators at 37 °C for 30 min in the dark, then washed thoroughly to remove extracellular dye, and subsequently de-esterified in calcium-free, phenol red-free DMEM at 37 °C. Fluorescence signals were acquired using Cytation 5 (BioTek, USA). Baseline fluorescence intensity (F0) was recorded continuously for 30 s prior to stimulation and defined as the mean fluorescence under resting conditions. Cells were then stimulated with 100 µM histamine to trigger IP3R-mediated ER calcium release, and fluorescence intensity (F) was recorded for a subsequent 120 s. Intracellular calcium mobilization was expressed as the ratio F/F0, representing the fold-change in fluorescence relative to the pre-stimulation baseline, and all values were normalized to those of the control group for inter-group comparisons [28].

### Co‑immunoprecipitation (Co‑IP)

Co-IP was performed using differentiated C2C12 myotubes to examine the physical interaction between GRP75 and VDAC1. Cells were lysed in ice-cold IP lysis buffer supplemented with protease inhibitor, and total protein concentrations were quantified using a BCA protein assay kit (Beyotime, China). For antibody-bead conjugation, an anti-GRP75 primary antibody was pre-coupled to Protein A/G magnetic beads by incubation at room temperature for 1.5 h, followed by three washes with PBS. The clarified cell lysates were then incubated with the antibody-bead complexes overnight at 4 °C. The following day, the immunoprecipitated complexes were washed three times with PBS to remove non-specifically bound proteins. Specifically co-precipitated proteins were eluted by boiling in SDS-PAGE loading buffer and analyzed by western blotting.

### Western blot analysis

To quantify protein expression levels, frozen muscle samples (30–40 mg) were homogenized and lysed in RIPA buffer supplemented with protease and phosphatase inhibitor cocktails. Total protein concentrations were determined using a BCA protein assay kit (Beyotime, China). Proteins were separated by SDS-PAGE on 7.5%-12% acrylamide gels and transferred onto nitrocellulose membranes by wet electrophoretic transfer. Membranes were blocked for 2 h at room temperature with 5% non-fat dry milk dissolved in TBS-Tween buffer and incubated at 4 °C overnight with primary antibodies. The following day, membranes were incubated with the corresponding secondary antibodies at room temperature for 1 h. Relative band intensities were quantified using ImageJ software, with target protein levels normalized to the corresponding loading control.

### Quantitative real-time PCR

Total RNA was extracted from TA and GAS muscles via tissue homogenization in TRIzol reagent and reverse transcribed via HiScript RT SuperMix following the manufacturer’s instructions. Quantitative real-time polymerase chain reaction (qRT-PCR) was performed via the CFX Connect Real-Time PCR System (CFX Manager, USA) with specific primers for MFN2 (forward primer: 5′-CAGACTATGCAGCAGCAGGACATGAT-3′, reverse primer: 5′-GAACTCGATGTCCTCCTGAAAGT-3′) and GAPDH (forward primer: 5′- GGTTGTCTCCTGCGACTTCA-3′, reverse primer: 5′- CCCTGTTGCTGTAGCCGTAT-3′). The results were normalized to the GAPDH levels via the 2^−△△Ct^ method of quantification to quantify the mRNA expression levels.

### RNA sequencing and data analysis

Total RNA was isolated and purified from the TA muscles of sham- and CLP-48 h mice according to the manufacturer’s protocol and sequenced at Aksomics Technology (Shanghai, China). The FPKMs of the gene and transcript levels were calculated via R software Ballgown. KEGG pathway and gene set enrichment analysis (GSEA) analyses were performed via customized programs (python/R/shell, etc.).

### Statistical analysis

All statistical analyses were performed using GraphPad Prism 10.1 (GraphPad Software, USA), and all data are presented as means ± SD. The specific definition of *n* is indicated in the corresponding figure legends. Prior to parametric testing, data normality was assessed using the Shapiro-Wilk test, and homogeneity of variances was confirmed using Levene’s test.

For comparisons between two independent groups, unpaired two-tailed Student’s t-tests were applied. For factorial experiments involving two independent variables, two-way ANOVA followed by Tukey’s honestly significant difference (HSD) post hoc test was applied to evaluate both main effects and their interaction. This design was applied to two categories of experiments: time-course experiments employing a 2 × 2 design with treatment condition (sham vs. CLP) and time point (24 h vs. 96 h) as the two independent variables; and AAV-mediated MFN2 overexpression experiments employing a 2 × 2 design with surgical condition (sham vs. CLP) and treatment (AAV-CON vs. AAV-MFN2) as the two independent variables. In AAV experiments where contralateral muscles from the same animal served as within-subject paired internal controls, the pairing structure was incorporated into the two-way ANOVA model as a blocking factor to account for inter-animal variability, and this pairing structure is explicitly noted in the corresponding figure legends. Pearson product–moment correlation coefficients were calculated to assess the strength and direction of linear associations between continuous normally distributed variables. For all analyses, a two-sided significance threshold of α = 0.05 was adopted, and *P*-values are reported throughout.

## Results

### Sepsis induces skeletal muscle atrophy in mice

To investigate the effects of sepsis on skeletal muscle, we established a murine sepsis model via CLP. Survival analysis demonstrated that CLP-operated mice exhibited progressive mortality, with survival rates of 80% at 24 h and 50% at 96 h post-surgery (Supplementary Fig. 1A), accompanied by significant reductions in body weight (Supplementary Fig. 1B). ELISA further confirmed markedly elevated serum concentrations of the pro-inflammatory cytokines IL-6, TNF-α, and IL-1β in septic mice (Supplementary Fig. 1C-E), collectively validating the successful establishment of the sepsis model.

**Fig. 1:**
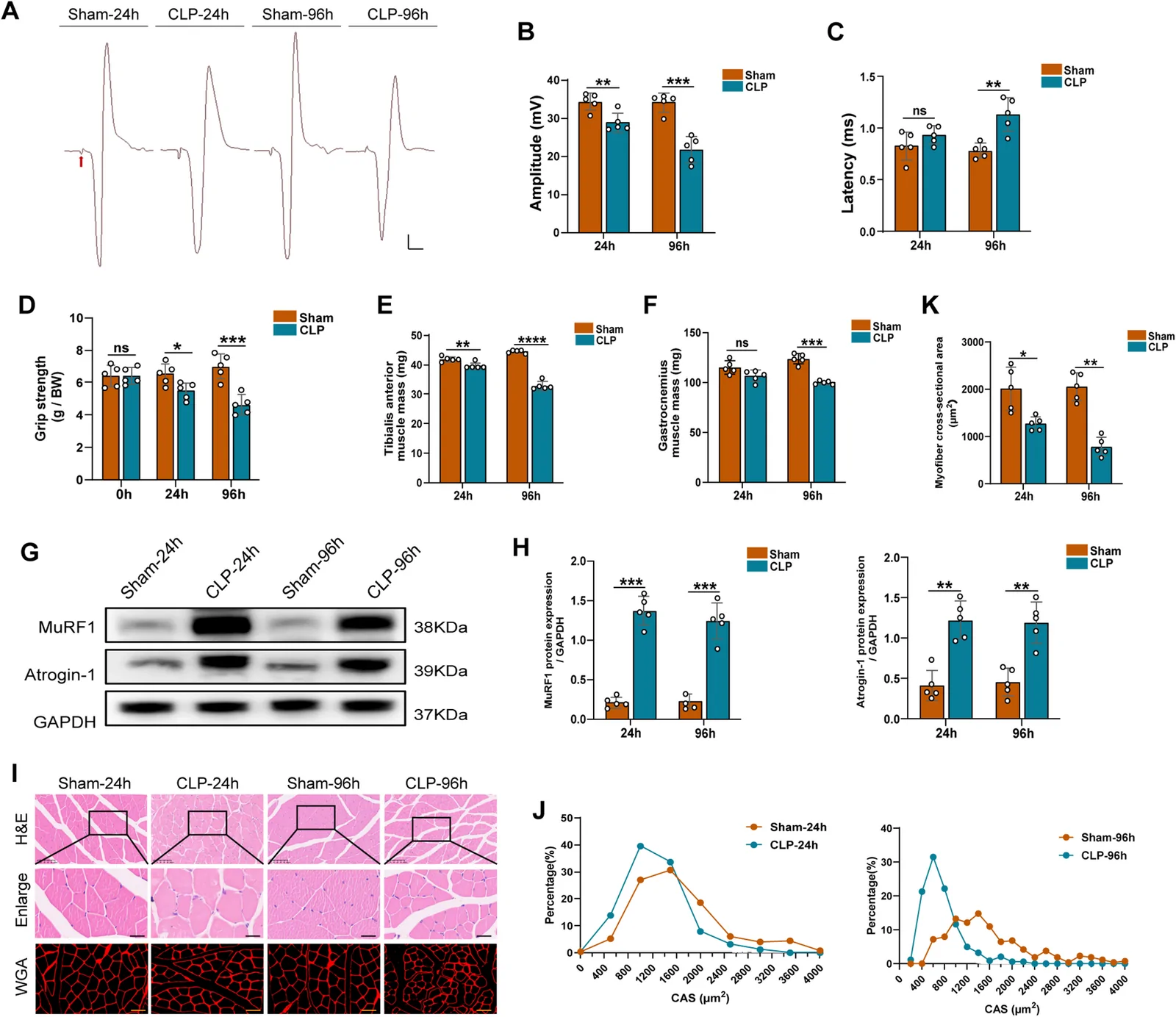
Sepsis induces skeletal muscle weakness and atrophy in mice. **A** Representative compound muscle action potential (CMAP) recordings of the lower limb muscles in sham and cecal ligation and puncture (CLP) mice, with quantitative analysis of (**B**) amplitude and (**C**) latency. **D** Quantitative assessment of forelimb grip strength normalized to body weight in sham and CLP mice. **E**-**F** Wet weights of the tibialis anterior (TA) and gastrocnemius (GAS) muscles following CLP or sham surgery. **G** Representative western blot images showing the protein expression of MuRF1 and Atrogin-1 in the TA muscle, with (**H**) quantitative analysis of MuRF1 and Atrogin-1 protein levels normalized to GAPDH. **I** Representative H&E and wheat germ agglutinin (WGA) staining images of GAS muscle cross-sections, with **J** frequency distribution histogram and (**K**) quantitative analysis of cross-sectional area (CSA) of muscle fibers. For panels A-D, n = 5 animals per group; for panels E-K, n = 5 muscles per group. All data are presented as means ± SD. Statistical comparisons across the 2 × 2 factorial design (model: sham vs. CLP; time point: 24 h vs. 96 h) were performed using two-way ANOVA followed by Tukey’s honestly significant difference (HSD) post hoc test. *P<0.05; **P<0.01; ***P<0.001; ****P<0.0001

To comprehensively characterize sepsis-induced skeletal muscle dysfunction, neuromuscular function was evaluated using CMAP recording and forelimb grip strength testing. CMAP analysis revealed a significant reduction in amplitude at CLP-24 h and a prolongation of latency at CLP-96 h (Fig. 1A-C), indicative of impaired neuromuscular transmission. Consistent with these findings, grip strength normalized to body weight was significantly decreased at CLP-24 h (Fig. 1D). To further quantify sepsis-induced muscle atrophy, the wet weights of the TA and GAS muscles were measured, and significant reductions were observed in TA muscle weight at CLP-24 h and in GAS muscle weight at CLP-96 h (Fig. 1E-F), indicating progressive muscle mass loss. At the molecular level, western blot analysis of the TA muscle revealed significantly upregulated protein expression of MuRF1 and Atrogin-1 beginning at CLP-24 h (Fig. 1G-H). Histological examination of GAS muscle cross-sections by H&E and WGA staining revealed marked disruption of muscle fiber morphology following CLP (Fig. 1I). Frequency distribution analysis of muscle fiber cross-sectional area (CSA) demonstrated a pronounced leftward shift toward smaller fiber diameters in CLP-operated mice (Fig. 1J), and quantitative analysis confirmed a significant reduction in mean CSA at CLP-24 h (Fig. 1K). Taken together, these findings demonstrate that CLP leads to progressive skeletal muscle atrophy in mice.

### Sepsis-induced mitophagy-associated remodeling in skeletal muscle is linked with mitochondrial calcium overload

**Fig. 2:**
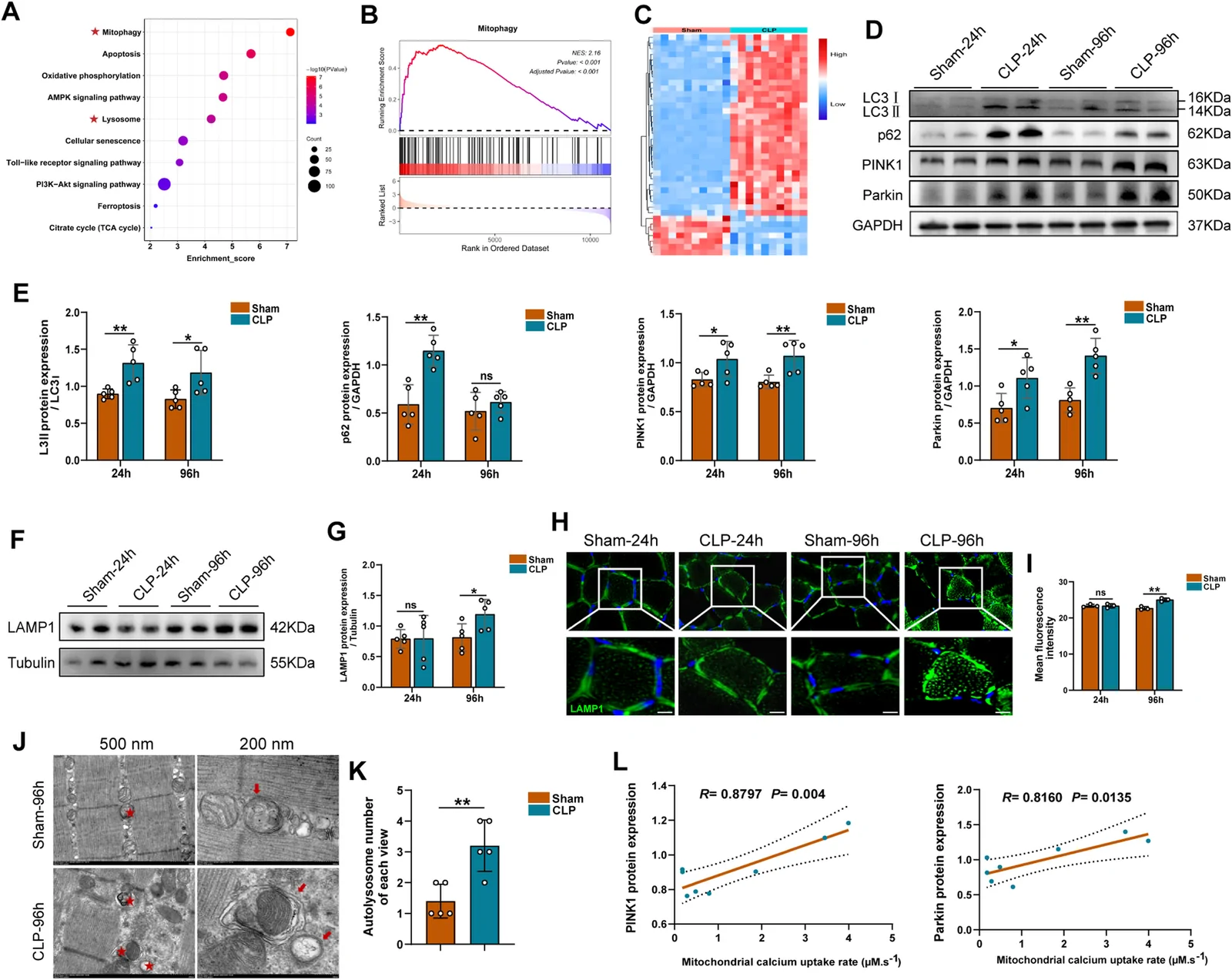
Sepsis-induced mitophagy-associated remodeling is associated with mitochondrial calcium overload in skeletal muscle. **A** KEGG pathway enrichment analysis of transcriptomic sequencing data highlighting significantly enriched pathways in septic skeletal muscle. **B** Gene set enrichment analysis (GSEA) plot illustrating the enrichment of mitophagy-related gene sets in the CLP group relative to the sham group. **C** Heatmap displaying the expression profiles of differentially expressed genes involved in mitophagy (*P* < 0.05); *n* = 10 animals per group. **D** Representative western blot images showing the protein expression of the mitophagy-related proteins LC3, p62, PINK1, and Parkin in the TA muscle, with (**E**) quantitative analysis of these protein levels normalized to GAPDH. **F** Representative western blot images showing the protein expression of the lysosomal membrane protein LAMP1 in the TA muscle, with (**G**) quantitative analysis of LAMP1 protein levels normalized to Tubulin. **H** Representative immunofluorescence images of LAMP1 in the TA muscle, with (**I**) quantitative analysis of mean LAMP1 fluorescence intensity, *n* = 3 muscles per group. **J** Representative transmission electron micrographs (TEM) illustrating mitophagosomes in the TA muscle, with (**K**) quantitative analysis of autophagosome number per field. **L** Pearson correlation analysis between mitochondrial calcium levels and the protein expression of PINK1 and Parkin. For panels **D**-**G** and **J**-**K**, *n* = 5 muscles per group. All data are presented as means ± SD. Statistical comparisons across the 2 × 2 factorial design (model: sham vs. CLP; time point: 24 h vs. 96 h) were performed using two-way ANOVA followed by Tukey’s HSD post hoc test. Pairwise correlations in panel **L** were assessed using Pearson product-moment correlation coefficients. *P<0.05; **P<0.01; ***P<0.001; ****P<0.0001

To investigate sepsis-mediated pathological remodeling in skeletal muscle, transcriptomic analyses were conducted on TA muscles isolated from sham and CLP-operated mice. KEGG pathway analysis revealed significant enrichment of mitophagy- and lysosome-related pathways in the CLP group (Fig. 2A), and subsequent GSEA demonstrated coordinated transcriptional upregulation of mitophagy-associated gene clusters (Fig. 2B). Heatmap analysis further confirmed that most mitophagy-related genes were upregulated in CLP-operated mice (Fig. 2C), collectively suggesting that SIM is associated with mitophagy.

To further characterize these transcriptomic findings at the protein level, we assessed the expression of mitophagy-associated markers in skeletal muscle. Western blot analysis revealed elevated protein levels of the autophagy-associated markers LC3 and p62, as well as the mitophagy-related proteins PINK1 and Parkin, at CLP-24 h (Fig. 2D-E). Notably, while p62 protein expression was significantly increased at CLP-24 h, no statistically significant change was observed at CLP-96 h (Fig. 2E). Given the temporal dissociation p62 dynamics expression, we hypothesized that lysosomal activity may undergo compensatory upregulation at later time points. Consistent with this hypothesis, protein expression of the lysosomal membrane protein LAMP1 was significantly elevated at CLP-96 h (Fig. 2F-G), a finding further corroborated by immunofluorescence analysis (Fig. 2H-I). TEM examination of TA muscle ultrastructure revealed a significantly greater number of mitochondria-containing autophagic vacuoles at CLP-96 h compared with sham group (Fig. 2J-K), providing morphological evidence consistent with mitophagy-associated structural remodeling.

Building upon our previous observations that sepsis-induced mitochondrial calcium overload contributes to skeletal muscle dysfunction [29], we examined the potential association between mitochondrial calcium levels and mitophagy-related protein expression. Pearson correlation analysis revealed significant positive associations between mitochondrial calcium overload intensity and the protein expression levels of both PINK1 (*R* = 0.8797, *P* < 0.01) and Parkin (*R* = 0.8160, *P* < 0.05) (Fig. 2L). These correlations suggest that mitochondrial calcium dysregulation may be associated with mitophagy-related signaling during sepsis-induced skeletal muscle atrophy, consistent with prior evidence from Song et al. [30] demonstrating a relationship between mitochondrial calcium overload and mitophagy-associated injury in cardiomyocytes. However, the precise mechanistic relationship between calcium overload and mitophagy-related remodeling in skeletal muscle warrants further investigation through interventional studies.

### The IP3R-GRP75-VDAC1 complex is associated with sepsis-induced mitochondrial calcium overload

**Fig. 3:**
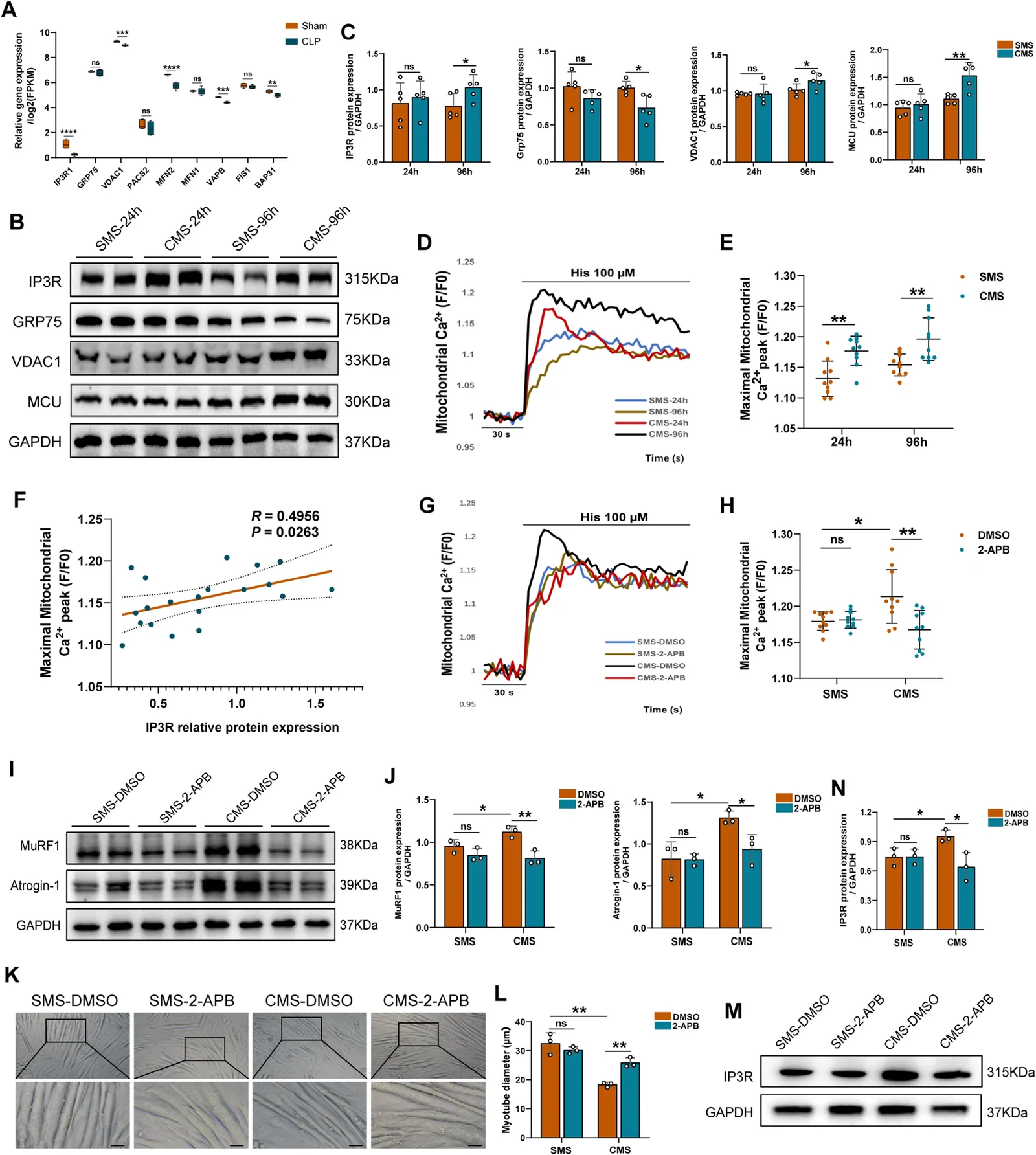
The IP3R–GRP75–VDAC1 complex is associated with sepsis-induced mitochondrial calcium overload in C2C12 myotubes. **A** RNA sequencing heatmap illustrating the relative expression levels of genes regulating ER-to-mitochondria calcium transport at the MAM in sham and CLP mice skeletal muscle. **B** Representative western blot images showing the protein expression of IP3R, GRP75, VDAC1, and MCU in the C2C12 myotubes, with (**C**) quantitative analysis of these protein levels normalized to GAPDH; *n* = 5 independent cell culture experiments. **D** Representative time-course fluorescence curves of Rhod-2 AM illustrating mitochondrial calcium uptake dynamics in C2C12 myotubes stimulated with 100 µM histamine, with (**E**) quantification of maximal mitochondrial calcium fluorescence intensity (F/F0); *n* = 10 independent wells per group. **F** Pearson correlation analysis between peak mitochondrial calcium levels and IP3R protein expression. **G** Representative time-course fluorescence curves of Rhod-2 AM illustrating mitochondrial calcium uptake dynamics in differentiated C2C12 myotubes following IP3R inhibition with 2-APB, with (**H**) quantification of maximal mitochondrial calcium fluorescence intensity (F/F0); *n* = 10 independent wells per group. **I** Representative western blot images showing the protein expression of MuRF1 and Atrogin-1 in C2C12 myotubes following 2-APB intervention, with (**J**) quantitative analysis of MuRF1 and Atrogin-1 protein levels normalized to GAPDH. **K** Representative bright-field microscopy images of differentiated C2C12 myotubes following 2-APB intervention, with (**L**) quantitative analysis of myotube diameters (30–40 myotubes measured per sample). **M** Representative western blot images confirming IP3R inhibition following treatment with 50 µM 2-APB in differentiated C2C12 myotubes, with (**N**) quantitative analysis of IP3R protein levels normalized to GAPDH. For panels **I**-**N**, *n* = 3 independent cell culture experiments. All data are presented as means ± SD. For panels **B**-**E** and **G**-**N**, statistical comparisons across the 2 × 2 factorial design (model: SMS vs. CMS; time point: 24 h vs. 96 h) were performed using two-way ANOVA followed by Tukey’s HSD post hoc test. Pairwise correlation in panel F was assessed using Pearson product-moment correlation coefficients. *P<0.05; **P<0.01; ***P<0.001; ****P<0.0001

Given that the ER serves as the primary intracellular calcium reservoir and the predominant source of mitochondrial calcium, the MAM represents a critical site for inter-organellar calcium communication [31]. Based on transcriptomic profiling of TA muscles, systematic analysis of MAM-associated calcium transport genes revealed significant downregulation of IP3R, VDAC1, MFN2, and VAPB at the mRNA level (Fig. 3A). To investigate whether the IP3R–GRP75–VDAC1 complex contributes to sepsis-induced mitochondrial calcium overload, C2C12 myotubes were treated with SMS or CMS to establish an in vitro model of sepsis [32]. Successful model establishment was confirmed by significantly elevated MuRF1 and Atrogin-1 protein expression and reduced myotube diameter at CMS-96 h (Supplementary Fig. 2A-D). Western blot analysis in CMS-treated myotubes revealed that IP3R, VDAC1, and MCU protein levels were elevated, whereas GRP75 expression was decreased (Fig. 3B-C). To validate these findings in vivo and address the discordance with RNA-seq data, we additionally assessed IP3R–GRP75–VDAC1 complex protein expression directly in the TA muscles of CLP-operated mice. Western blot analysis confirmed that at CLP-96 h, IP3R, VDAC1, and MCU protein levels were significantly elevated, while GRP75 was decreased relative to sham groups (Supplementary Fig. 3A-E), consistent with the cell model findings. The discordance between mRNA downregulation and protein upregulation likely reflects the complex post-transcriptional and translational regulatory mechanisms governing gene expression, compounded by dynamic shifts in protein synthesis and degradation as sepsis transitions from the acute inflammatory to the recovery phase in skeletal muscle [33].

To assess the functional consequences of IP3R activity, CMS-treated C2C12 myotubes were stimulated with 100 µM histamine to trigger IP3R-mediated calcium release, and mitochondrial calcium uptake was monitored using Rhod-2 AM fluorescence (Fig. 3D). CMS treatment progressively increased mitochondrial calcium concentration, with further elevation observed at CMS-96 h compared with CMS-24 h (Fig. 3E), indicating that sepsis promotes sustained mitochondrial calcium overload. Pearson correlation analysis further revealed a significant positive association between IP3R protein expression and mitochondrial calcium levels (*R* = 0.4956, *P* = 0.0263; Fig. 3F), suggesting that IP3R abundance is associated with the degree of mitochondrial calcium overload.

To pharmacologically evaluate the role of IP3R-mediated calcium transfer, CMS-treated C2C12 myotubes were treated with 50 µM 2-APB, a selective IP3R channel inhibitor. 2-APB treatment significantly attenuated mitochondrial calcium uptake in CMS-treated myotubes (Fig. 3G-H), providing pharmacological support for the role of IP3R-dependent calcium transfer in sepsis-induced mitochondrial calcium overload. At the molecular level, 2-APB co-treatment substantially reversed CMS-induced upregulation of MuRF1 and Atrogin-1 protein expression (Fig. 3I-J) and restored myotube diameter (Fig. 3K-L). Additionally, consistent with prior reports that 2-APB can reduce IP3R protein abundance beyond its channel-blocking activity [34, 35], western blot analysis revealed that 2-APB also attenuated CMS-induced IP3R protein upregulation (Fig. 3M-N). Collectively, these data indicate that IP3R-mediated calcium transfer at MAM is associated with mitochondrial calcium overload and atrophic remodeling in CMS-treated myotubes.

### Sepsis downregulates MFN2 expression and reduces ER-mitochondria distance in skeletal muscle

**Fig. 4:**
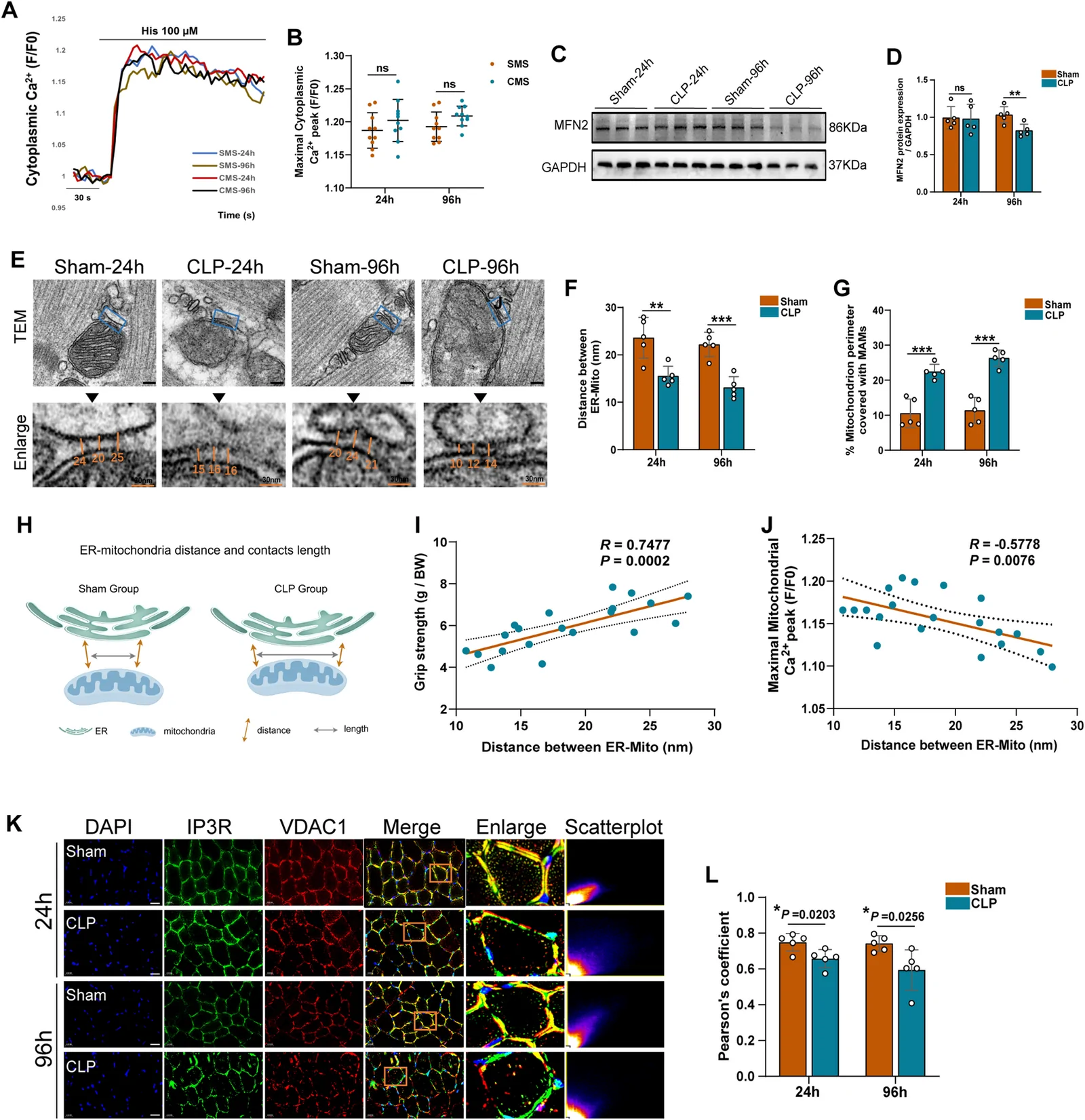
Sepsis downregulates MFN2 expression and reduces ER–mitochondria distance in skeletal muscle. **A** Representative time-course fluorescence curves of Fluo-4 AM illustrating cytosolic calcium dynamics in differentiated C2C12 myotubes stimulated with 100 µM histamine, with (**B**) quantification of maximal cytosolic calcium fluorescence intensity (F/F0); n = 10 independent wells per group. **C** Representative western blot images showing MFN2 protein expression in the TA muscle, with (**D**) quantitative analysis of MFN2 protein levels normalized to GAPDH. **E** Representative TEM illustrating MAM ultrastructure in the TA muscle (scale bar: 30 nm), with (**F**) quantitative analysis of ER-mitochondria distances and (**G**) the ratio of MAM interface length to total mitochondrial perimeter. **H** Schematic illustration depicting the measurement parameters of ER-mitochondria contact points, including inter-membrane distance and MAM interface length. **I** Pearson correlation analysis between ER-mitochondria distance and forelimb grip strength in septic mice, and (**J**) Pearson correlation analysis between ER-mitochondria distance and peak mitochondrial calcium levels in differentiated C2C12 myotubes. **K** Representative fluorescence images of IP3R–VDAC1 colocalization in the TA muscle, with (**L**) quantitative analysis of PCC. For panels C-G and K-L, n = 5 muscles per group. All data are presented as means ± SD. Statistical comparisons across the 2 × 2 factorial design (model: sham vs. CLP; time point: 24 h vs. 96 h) for panels C-G and K-L were performed using two-way ANOVA followed by Tukey’s HSD post hoc test. Pairwise correlations in panels I and J were assessed using Pearson product-moment correlation coefficients. *P<0.05; **P<0.01; ***P<0.001; ****P<0.0001

The transcriptomic analysis described above identified significant alterations in MFN2 and VAPB gene expression in addition to the IP3R–GRP75–VDAC1 complex (Fig. 3A), implicating these MAM-associated proteins in sepsis-induced mitochondrial calcium dysregulation. To determine whether the observed increase in mitochondrial calcium uptake was accompanied by changes in cytosolic calcium, we monitored IP3R-mediated cytosolic calcium dynamics using Fluo-4 AM (Fig. 4A). Notably, no statistically significant differences in cytosolic calcium levels were detected among groups (Fig. 4B), despite the significant elevation of mitochondrial calcium uptake in CMS-treated myotubes (Fig. 3D-E). This selective increase in mitochondrial, but not cytosolic, calcium accumulation suggested that structural alterations at the ER-mitochondria interface, leading us to investigate MAM structure and the ER-mitochondria distance.

Given the essential role of MFN2 in maintaining optimal ER-mitochondria distance and facilitating inter-organellar calcium transfer [16], we assessed MFN2 protein expression in the TA muscles of CLP-operated mice by western blotting. The results showed MFN2 protein levels were significantly decreased at CLP-96 h (Fig. 4C-D). TEM analysis of TA muscle ultrastructure revealed a significant reduction in ER-mitochondria distance in CLP group (Fig. 4E-F), accompanied by a significant increase in the ratio of MAM interface length to total mitochondrial perimeter (Fig. 4G), as schematically illustrated in Fig. 4H. Pearson correlation analyses further demonstrated that the ER-mitochondria distance was significantly positively correlated with forelimb grip strength (Fig. 4I) and significantly negatively correlated with peak mitochondrial calcium levels in CMS-treated C2C12 myotubes (Fig. 4J), collectively suggesting that MFN2-mediated regulation of ER-mitochondria distance is associated with both skeletal muscle function and mitochondrial calcium homeostasis.

To further characterize reduced ER-mitochondria distance on MAM integrity, immunofluorescence co-staining of IP3R and VDAC1 was performed in TA muscle. Unexpectedly, CLP-operated mice exhibited significantly reduced IP3R–VDAC1 colocalization (Fig. 4K-L), which appeared paradoxical given the observed reduction in physical distance by TEM. This apparent discrepancy may be explained by the concurrent downregulation of MFN2, as a mitochondrial fusion protein, may alter mitochondrial morphology and network organization, thereby indirectly affecting fluorescence colocalization [36].

### MFN2 overexpression increases ER-mitochondria distance and alleviates mitochondrial calcium overload in septic skeletal muscle

**Fig. 5:**
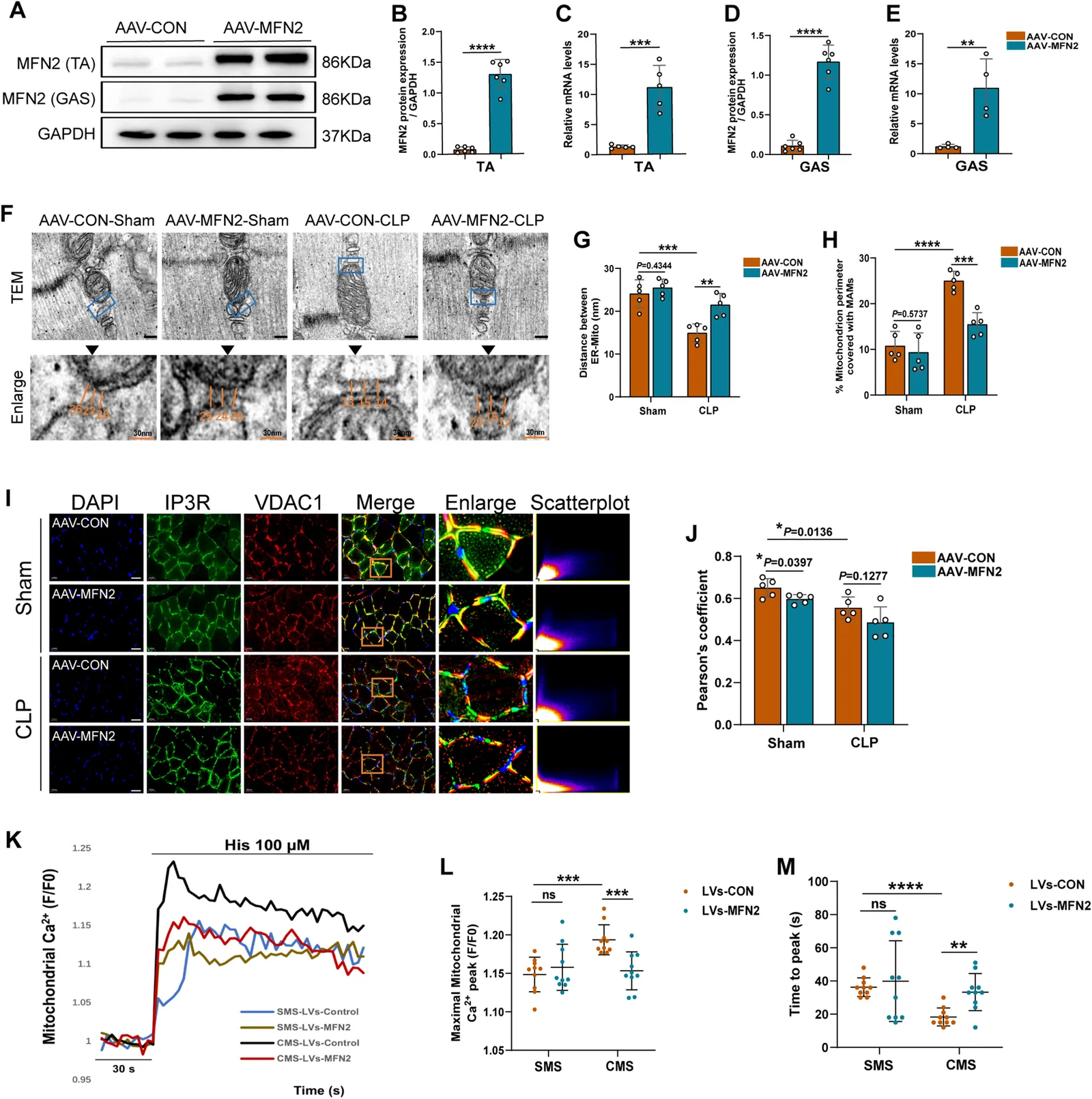
MFN2 overexpression increased ER-mitochondria distance and alleviated mitochondrial calcium overload in the skeletal muscle of septic mice. **A** Representative western blot images confirming MFN2 overexpression in the TA and GAS muscles following AAV injection, with (**B**-**E**) quantitative analysis of MFN2 protein expression normalized to GAPDH and MFN2 mRNA levels by qPCR. **F** Representative TEM illustrating MAM ultrastructure in the TA muscle (scale bar: 30 nm), with (**G**) quantitative analysis of ER-mitochondria distance and (**H**) the ratio of MAM interface length to total mitochondrial perimeter. **I** Representative fluorescence images of IP3R–VDAC1 colocalization in the TA muscle, with (**J**) quantitative analysis of Pearson’s colocalization coefficients. **K** Representative time-course fluorescence curves of Rhod-2 AM illustrating mitochondrial calcium uptake dynamics in differentiated C2C12 myotubes following MFN2 overexpression and stimulation with 100 µM histamine, with (**L**) quantification of maximal mitochondrial calcium fluorescence intensity (F/F0) and (**M**) statistical analysis of time to peak fluorescence. For panels A-J, n = 5 muscles per group; for panels K-M, n = 10 independent wells per group. All data are presented as means ± SD. For panels A-J, contralateral muscles from the same animal injected with AAV-CON served as within-subject paired internal controls; n = 5 animals per group, with each animal contributing one AAV-MFN2 muscle and one contralateral AAV-CON muscle (paired design). Statistical comparisons across the 2 × 2 factorial design (model: sham vs. CLP; treatment: AAV-CON vs. AAV-MFN2) were performed using two-way ANOVA followed by Tukey’s HSD post hoc test; the within-subject pairing structure was incorporated as a blocking factor to account for inter-animal variability. For panels K–M, statistical comparisons across the 2 × 2 factorial design (model: SMS vs. CMS; treatment: LVs-CON vs. LVs-MFN2) were performed using two-way ANOVA followed by Tukey’s HSD post hoc test. *P<0.05; **P<0.01; ***P<0.001; ****P<0.0001

To directly investigate the role of MFN2 in regulating mitochondrial calcium homeostasis in septic skeletal muscle, AAV was used to overexpress MFN2 in the TA and GAS muscles of mice, and lentiviral transduction was performed in C2C12 myoblasts. Successful MFN2 overexpression was confirmed at both the protein and mRNA levels by western blotting and qPCR in TA muscles (Fig. 5A-C), GAS muscles (Fig. 5D-E), and C2C12 myoblasts (Supplementary Fig. 4A-B). TEM analysis demonstrated that MFN2 overexpression significantly increased the ER-mitochondria distance and reduced the ratio of MAM interface length to total mitochondrial perimeter (Fig. 5F-H), confirming that MFN2 regulates ER-mitochondria structure in skeletal muscle.

Immunofluorescence co-staining of IP3R and VDAC1 was subsequently performed in TA muscle to assess the effect of MFN2 overexpression on inter-organellar colocalization (Fig. 5I). Consistent with the TEM findings, AAV-MFN2 injection in sham-operated mice significantly reduced IP3R and VDAC1 fluorescence colocalization compared with AAV-CON-Sham group (*P* = 0.0397; Fig. 5J), indicating that increased ER-mitochondria distance attenuates fluorescence colocalization between the two organelles. CLP also significantly reduced colocalization in AAV-CON mice compared with sham group (*P* = 0.0136). The difference between the AAV-MFN2-CLP and AAV-CON-CLP groups did not reach statistical significance (*P* = 0.1277), suggesting that MFN2 overexpression may partially rescue sepsis-induced disruption of mitochondrial network organization.

To assess the functional consequences of MFN2-mediated changes in ER-mitochondria distance on calcium dynamics, MFN2-overexpressing C2C12 myotubes were treated with CMS for 96 h, and mitochondrial calcium uptake was monitored following histamine-induced IP3R activation (Fig. 5K). MFN2 overexpression significantly attenuated mitochondrial calcium overload in CMS-treated myotubes compared with LVs-CON controls (Fig. 5L). Furthermore, analysis of calcium transfer kinetics revealed that MFN2 overexpression significantly prolonged the time to peak mitochondrial calcium following SR calcium release (Fig. 5M), suggesting that increasing ER-mitochondria distance reduces the efficiency of ER-to-mitochondria calcium transfer. Collectively, these findings demonstrate that sepsis-induced MFN2 downregulation reduces ER-mitochondria distance, thereby facilitating excessive ER-to-mitochondria calcium transfer and contributing to mitochondrial calcium overload in skeletal muscle. Restoration of MFN2 expression via AAV-mediated overexpression reverses this structural remodeling and mitigates mitochondrial calcium overload.

### MFN2 overexpression alleviates sepsis-induced skeletal muscle atrophy in vivo and in vitro

**Fig. 6:**
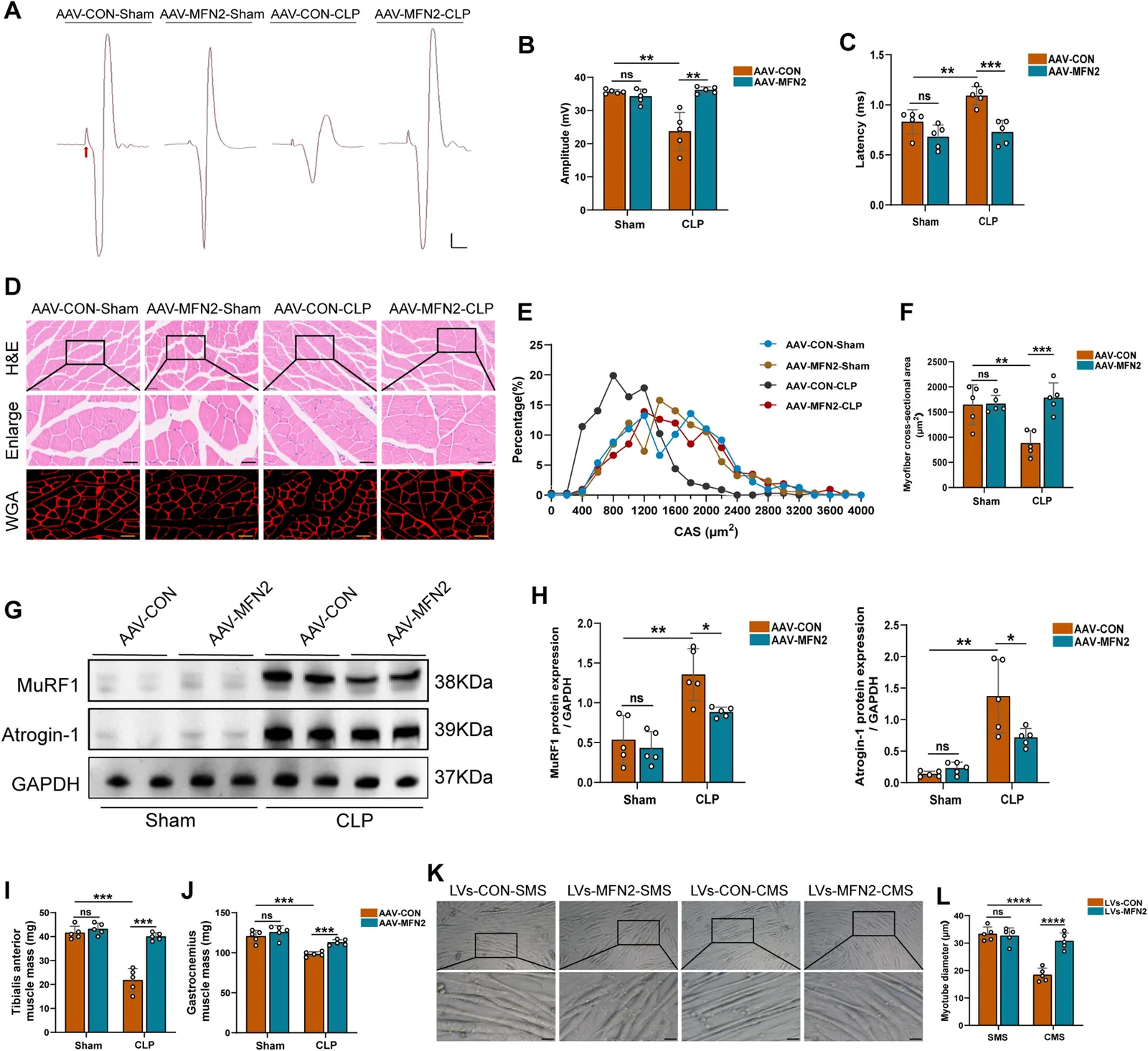
MFN2 overexpression alleviates sepsis-induced skeletal muscle atrophy in vivo and in vitro. **A** Representative CMAP recordings in mice following AAV-mediated MFN2 overexpression in the TA and GAS muscles, with quantitative analysis of (**B**) CMAP amplitude and (**C**) CMAP latency. **D** Representative H&E and WGA staining images of GAS muscle cross-sections following MFN2 overexpression, with (**E**) frequency distribution histogram and (**F**) quantitative analysis of CSA of muscle fibers. **G** Representative western blot images showing the protein expression of MuRF1 and Atrogin-1 in TA muscles following MFN2 overexpression, with (**H**) quantitative analysis normalized to GAPDH. **I**-**J** Wet weights of the TA and GAS muscles following AAV-MFN2 intervention. **K** Representative bright-field microscopy images of differentiated C2C12 myotubes following MFN2 overexpression, with (**L**) quantitative analysis of myotube diameters (30–40 myotubes measured per sample). For panels A-C, *n* = 5 animals per group; for panels D-J, *n* = 5 muscles per group; for panels K-L, *n* = 5 independent cell culture experiments. All data are presented as means ± SD. For panels A-J, contralateral muscles from the same animal injected with AAV-CON served as within-subject paired internal controls; *n* = 5 animals per group, with each animal contributing one AAV-MFN2 muscle and one contralateral AAV-CON muscle (paired design). Statistical comparisons across the 2 × 2 factorial design (model: sham vs. CLP; treatment: AAV-CON vs. AAV-MFN2) were performed using two-way ANOVA followed by Tukey’s HSD post hoc test; the within-subject pairing structure was incorporated as a blocking factor to account for inter-animal variability. For panels K-L, statistical comparisons across the 2 × 2 factorial design (model: SMS vs. CMS; treatment: LVs-CON vs. LVs-MFN2) were performed using two-way ANOVA followed by Tukey’s HSD post hoc test. *P<0.05; **P<0.01; ***P<0.001; ****P<0.0001

To investigate whether MFN2 overexpression can mitigate SIM by alleviating mitochondrial calcium overload, neuromuscular function was assessed by CMAP recording in AAV-injected mice (Fig. 6A). The results showed that MFN2 overexpression significantly increased CMAP amplitude and shortened latency in AAV-MFN2-CLP mice compared with AAV-CON-CLP mice (Fig. 6B-C). Histological analysis of GAS muscle cross-sections by H&E and WGA staining revealed that CLP-induced reductions in muscle fiber CSA were substantially attenuated by MFN2 overexpression (Fig. 6D-F). Notably, the frequency distribution of muscle fiber CSA in the AAV-MFN2-CLP group closely approximated that of the AAV-CON-Sham group (Fig. 6E). At the molecular level, western blot analysis of TA muscles demonstrated that CLP-induced upregulation of MuRF1 and Atrogin-1 was significantly reversed by MFN2 overexpression (Fig. 6G-H). Furthermore, MFN2 overexpression significantly attenuated CLP-induced reductions in both TA and GAS muscle wet weights (Fig. 6I-J), demonstrating preservation of muscle mass in vivo.

Complementary in vitro analysis in MFN2-overexpressing C2C12 myotubes confirmed that CMS-induced reductions in myotube diameter were significantly alleviated by MFN2 overexpression compared with LVs-CON controls (Fig. 6K-L). Collectively, these findings demonstrate that AAV-mediated MFN2 overexpression effectively alleviates sepsis-induced skeletal muscle dysfunction and atrophy both in vivo and in vitro.

### MFN2 overexpression modulates GRP75–VDAC1 interaction to attenuate mitochondrial calcium overload

The findings described above revealed that sepsis-induced mitochondrial calcium overload in skeletal muscle was associated with concurrent alterations in both MFN2 expression and IP3R–GRP75–VDAC1 complex protein abundance. Given that MAM-associated proteins have been shown to regulate IP3R–GRP75–VDAC1 complex assembly [21, 37], we investigated whether MFN2 influences mitochondrial calcium homeostasis through mechanisms by modulating the expression and assembly of the IP3R–GRP75–VDAC1 complex beyond regulation of ER-mitochondria distance.

**Fig. 7:**
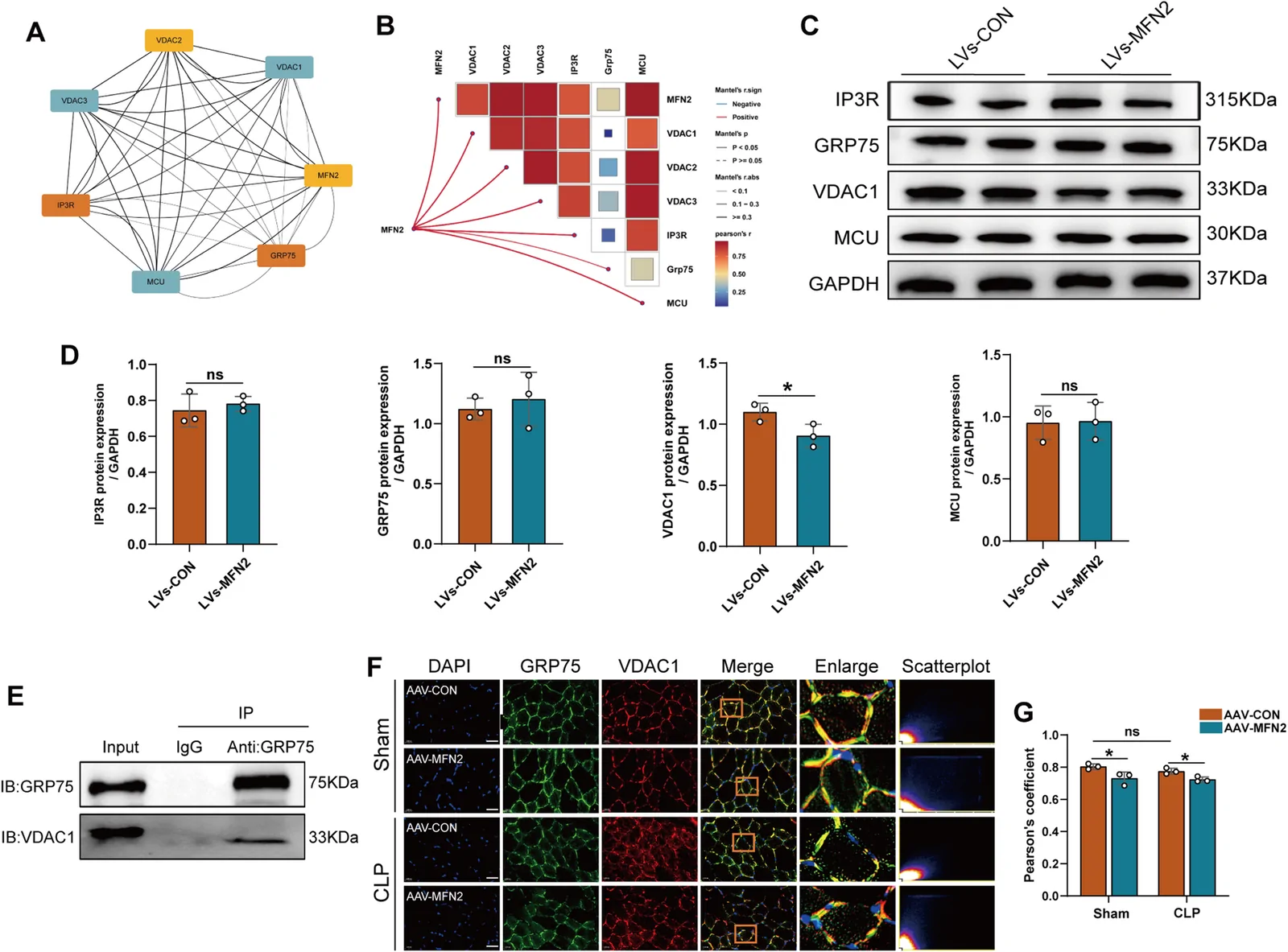
MFN2 overexpression modulates GRP75–VDAC1 interaction to attenuate mitochondrial calcium overload. **A** Differential gene co-expression network map illustrating the regulatory relationships between MFN2 and key components of the IP3R-GRP75-VDAC1/MCU axis. **B** Correlation heatmap depicting pairwise PCC among MFN2, IP3R, GRP75, VDAC1, and MCU expression levels; color intensity represents the magnitude and direction of correlation. **C** Representative western blot images showing the protein expression levels of IP3R, GRP75, VDAC1, and MCU in differentiated C2C12 myotubes following MFN2 overexpression, with (**D**) quantitative analysis of each target protein normalized to GAPDH. **E** Co-immunoprecipitation analysis using an anti-GRP75 antibody demonstrating the physical interaction between GRP75 and VDAC1 in differentiated C2C12 myotubes following MFN2 overexpression and immunoprecipitated VDAC1 was detected by western blotting. **F** Representative confocal fluorescence images illustrating the spatial colocalization of GRP75 and VDAC1 in the TA muscles of mice following AAV-mediated MFN2 overexpression, with (**G**) quantitative analysis of Pearson product-moment correlation coefficients. For panels C-D, *n* = 3 independent cell culture experiments; for panels F-G, *n* = 3 muscles per group. All data are presented as means ± SD. For panels C-D, between-group comparisons were performed using unpaired two-tailed Student’s t-tests. For panels F-G, contralateral muscles from the same animal injected with AAV-CON served as within-subject paired internal controls; *n* = 3 animals per group, with each animal contributing one AAV-MFN2 muscle and one contralateral AAV-CON muscle (paired design). Statistical comparisons across the 2 × 2 factorial design (model: sham vs. CLP; treatment: AAV-CON vs. AAV-MFN2) were performed using two-way ANOVA followed by Tukey’s HSD post hoc test; the within-subject pairing structure was incorporated as a blocking factor to account for inter-animal variability. *P**<**0.05; **P**<**0.01; ***P**<**0.001; ****P**<**0.0001

To explore the potential transcriptional relationship between MFN2 and complex components, a differential gene co-expression network was constructed using Cytoscape software, and pairwise correlation analysis was performed on transcriptomic data using OmicStudio tools. These analyses revealed significant transcriptional co-regulation between MFN2 and both IP3R and VDAC1 at the gene expression level (Fig. 7A-B), providing preliminary evidence for a functional relationship between MFN2 and the IP3R-GRP75-VDAC1/MCU axis. To investigate whether changes in MFN2 protein abundance influence the expression of individual complex components, we assessed the protein levels of IP3R, GRP75, VDAC1, and MCU in MFN2-overexpressing TA muscles and C2C12 myotubes. MFN2 overexpression selectively decreased VDAC1 protein expression without significantly affecting IP3R, GRP75, or MCU levels, both in C2C12 myotubes (Fig. 7C-D) and in TA muscles (Supplementary Fig. 4C-D). These findings suggest that MFN2 may contribute to the modulation of IP3R–GRP75–VDAC1 complex assembly, at least in part, through downregulation of VDAC1 protein abundance.

Given that GRP75 functions as structural bridge between IP3R on the ER membrane and VDAC1 on the OMM, thereby facilitating inter-organellar calcium transfer [38], we next investigated whether MFN2-mediated reduction in VDAC1 expression is associated with altered GRP75–VDAC1 protein interaction. Co-IP analysis using an anti-GRP75 antibody confirmed a physical interaction between GRP75 and VDAC1 in C2C12 myotubes (Fig. 7E). Immunofluorescence co-staining further revealed that GRP75–VDAC1 spatial colocalization was significantly reduced in the AAV-MFN2-CLP group compared with the AAV-CON-CLP group (Fig. 7F-G), suggesting that MFN2 overexpression is associated with attenuated GRP75–VDAC1 interaction in septic skeletal muscle. Taken together, these findings suggest that MFN2 overexpression modulates GRP75–VDAC1 interaction and IP3R–GRP75–VDAC1 complex assembly, and that this modulation may contribute to the attenuation of ER-to-mitochondria calcium transfer and mitochondrial calcium overload in SIM.

## Discussion

Muscle weakness is a common and debilitating complication of sepsis, affecting 70–100% of survivors of severe sepsis and contributing to increased long-term mortality [39]. Current evidence identifies three principal mechanisms underlying SIM: mitochondrial dysfunction, enhanced muscle proteolysis, and impaired muscle excitability [1]. Kingren et al. [40] reported that mitochondrial abnormalities progressively worsened from the acute to the chronic phase of sepsis, and that mice recovering muscle mass but retaining chronic weakness exhibited persistent mitochondrial dysfunction, underscoring the central role of mitochondrial pathology in SIM.

Building on our previous identification of mitochondrial calcium overload as a key mechanism in SIM [29], the present study investigated the upstream structural and molecular determinants of this pathological process at the MAM. As the primary intracellular calcium reservoir and the predominant source of mitochondrial calcium, the ER exerts its influence on mitochondrial calcium homeostasis largely through the spatial organization of MAM [41]. The IP3R–GRP75–VDAC1 complex functions as a quasi-synaptic calcium transfer unit at the MAM, and its upregulation has been shown to promote mitochondrial calcium overload and apoptosis in bovine mammary epithelial cells exposed to lipopolysaccharide [34]. In our study, sepsis induced elevated protein expression of IP3R, VDAC1, and MCU, alongside a decrease in GRP75 in C2C12 myotubes. Given that GRP75 serves as the structural bridge of the IP3R–GRP75–VDAC1 complex, its downregulation may represent a compensatory response aimed at limiting excessive ER-to-mitochondria calcium transfer [38, 42]. It has also been reported that inhibiting IP3R can alleviate vanadium-induced mitochondrial calcium overload in hepatocytes and reduce inflammatory responses by downregulating the IP3R-GRP75-VDAC1/MCU axis [43]. In our study, pharmacological inhibition of IP3R with 2-APB attenuated mitochondrial calcium uptake and ameliorated CMS-induced muscle atrophy in C2C12 myotubes, providing pharmacological support for a role of IP3R-dependent calcium transfer in this process. However, it should be noted that 2-APB was also observed to reduce IP3R protein abundance in addition to its channel-blocking activity, which precludes definitive dissociation of these two effects.

MFN2, the first identified MAM-tethering protein, plays a critical role in maintaining ER-mitochondria contact [44]. Notably, subsequent studies have demonstrated that reduced MFN2 expression can paradoxically enhance MAM junction formation and promote mitochondrial calcium overload [30, 45], consistent with our observation that sepsis-induced MFN2 downregulation reduced ER-mitochondria distance and contributed to mitochondrial calcium overload. These apparently contradictory effects of MFN2 on MAM structure likely reflect context-dependent differences across disease models and organ systems. Regarding the discrepancy between reduced fluorescence colocalization and reduced ER-mitochondria distance observed in our study, this is consistent with the findings of Filadi et al. [19], who similarly reported reduced fluorescence colocalization despite closer inter-organellar proximity by electron microscopy following MFN2 downregulation. In a previous study, we demonstrated that CLP at day 5 post-induction was associated with increased mitochondrial fission and reduced fusion in TA muscles, resulting in significant mitochondrial morphological remodeling [46]. As MFN2 is a critical regulator of mitochondrial fusion, its downregulation likely disrupts mitochondrial network organization, which may reduce apparent fluorescence colocalization independently of actual physical contact distance [36, 47]. These observations highlight an important methodological consideration: in disease models characterized by MFN2-dependent mitochondrial morphological remodeling, fluorescence colocalization should be regarded as a morphology-influenced surrogate readout rather than a direct structural indicator of MAM integrity, and TEM-based measurement of ER-mitochondria distance should be considered the more reliable indicator of MAM structure in this context.

The IP3R–GRP75–VDAC1 complex is regulated by various MAM-associated proteins, including IRE1α, DJ-1, and FUNDC1 [21]. Han et al. [48] demonstrated that MFN2 knockdown in hippocampal pyramidal neurons decreased IP3R-GRP75 interaction and attenuated mitochondrial calcium uptake, suggesting that MFN2 may modulate IP3R–GRP75–VDAC1 complex assembly. In the present study, MFN2 overexpression selectively decreased VDAC1 protein expression without significantly affecting IP3R, GRP75, or MCU levels. Co-IP analysis confirmed a physical interaction between GRP75 and VDAC1, and immunofluorescence analysis revealed that GRP75–VDAC1 spatial colocalization was significantly reduced following MFN2 overexpression in CLP-operated mice. Taken together, these findings suggest that MFN2 overexpression modulates GRP75–VDAC1 interaction and IP3R–GRP75–VDAC1 complex assembly, likely through VDAC1 downregulation, and that this modulation may contribute to attenuated ER-to-mitochondria calcium transfer in septic skeletal muscle. However, it should be noted that the precise molecular mechanism by which MFN2 regulates VDAC1 protein abundance and GRP75–VDAC1 interaction warrants further investigation. In addition, whether MFN2 additionally regulates other MAM-tethering complexes, such as VAPB-PTPIP51 or BAP31-FIS1, remains to be investigated.

## Conclusion

**Fig. 8:**
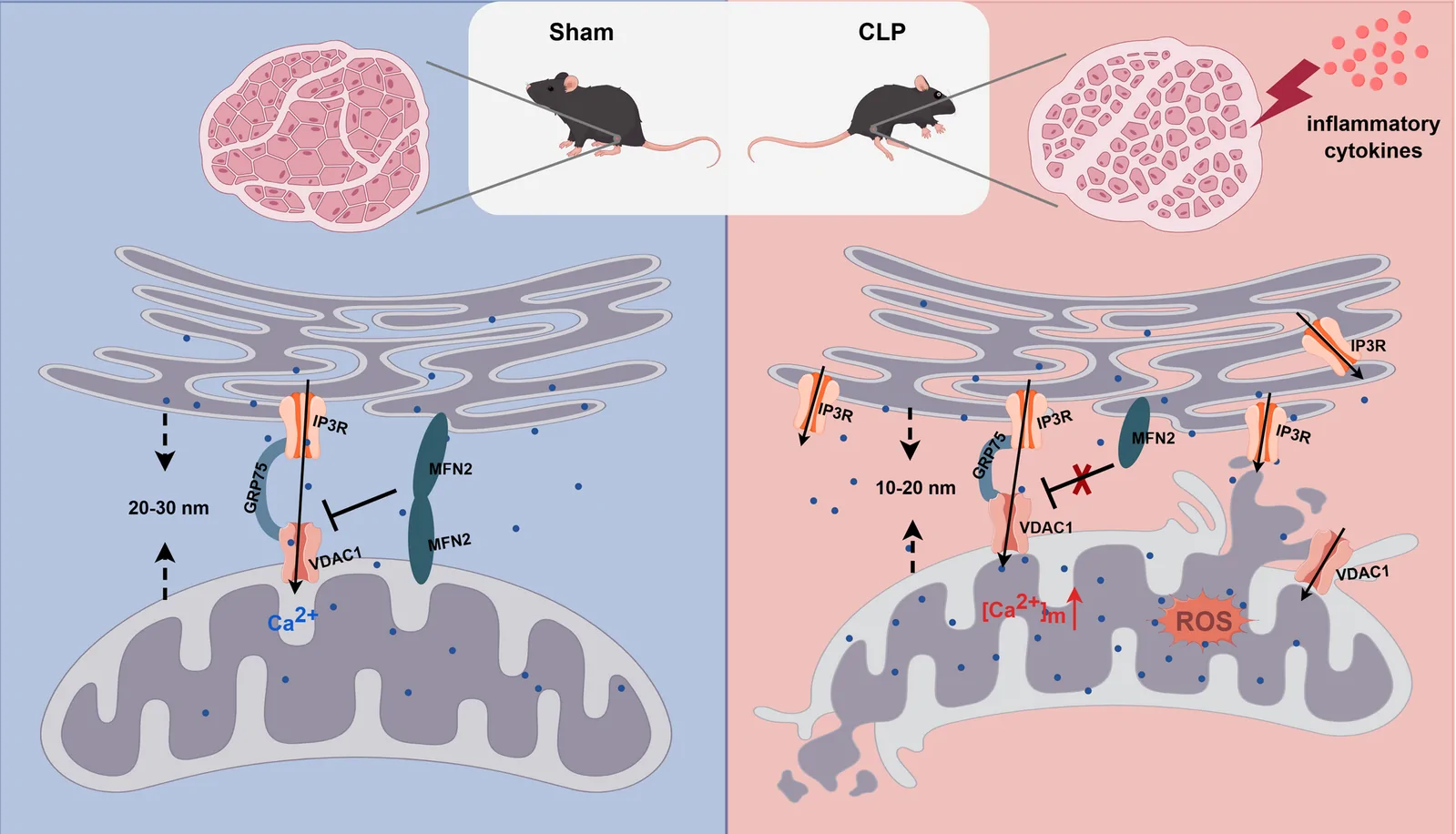
The mechanism diagram of the study. Under physiological conditions, MFN2 forms homodimers at MAM that maintain the ER-mitochondrion distance within the range of 20–30 nm, supporting regulated calcium transfer from the ER to the mitochondrial via the IP3R–GRP75–VDAC1 complex, thereby preserving mitochondrial calcium homeostasis. During sepsis, MFN2 protein expression is significantly downregulated in skeletal muscle, shortening the ER-mitochondrion distance to approximately 10–20 nm and directly facilitating calcium from the ER to mitochondria. Concurrently, upregulation of IP3R and VDAC1 protein expression promotes IP3R–GRP75–VDAC1 complex-mediated calcium transfer. The combined effects lead to mitochondrial calcium overload, which is subsequently associated with mitochondrial membrane disruption, morphological abnormalities, and skeletal muscle fiber atrophy

Our findings demonstrate that SIM is associated with mitochondrial calcium overload driven by sepsis-induced MFN2 downregulation. MFN2 overexpression attenuates this pathological process through two complementary mechanisms, including restoring ER-mitochondria distance and modulating GRP75–VDAC1 interaction. These effects collectively contribute to the amelioration of skeletal muscle atrophy in septic mice (Fig. 8).

## Supplementary Information

Supplementary Material 1.

Supplementary Material 2.

Supplementary Material 3.

Supplementary Material 4.

## Data Availability

The data analyzed during this study are included in this published article and the supplemental data files. Additional supporting data are available from the corresponding authors upon reasonable request.

## Abbreviations

SIM: Sepsis-induced myopathy
MAM: Mitochondria-associated endoplasmic reticulum membrane
SR: Sarcoplasmic reticulum
ER: Endoplasmic reticulum
CLP: Cecal ligation and puncture
2-APB: 2-Aminoethyl diphenylborinate
CMAP: Compound muscle action potential
TA: Tibialis anterior
GAS: Gastrocnemius
AAV: Adeno-associated virus
LV: Lentivector
CSA: Cross-sectional area
MFN2: Mitofusin 2
IP3R: Inositol 1,4,5-trisphosphate receptor
GRP75: Glucose-regulated protein 75
VDAC1: Voltage-dependent anion channel 1
MCU: Mitochondrial calcium uniporter
MuRF1: Muscle-specific RING finger protein 1
Atrogin-1: Muscle atrophy gene-1
WGA: Wheat germ agglutinin
Co-IP: Co-immunoprecipitation
PINK1: PTEN-induced putative kinase 1
LAMP1: Lysosomal-Associated Membrane Protein 1
