# Supplementary material for: MFN2 alleviates mitochondrial calcium overload in sepsis-induced myopathy by modulating GRP75–VDAC1 interaction
Source: Skelet Muscle. 2026 Aug 13;16:41. doi: 10.1186/s13395-026-00441-6 (PMC13749854; doi:10.1186/s13395-026-00441-6)

Supplemental Materials to Fig. 1 G

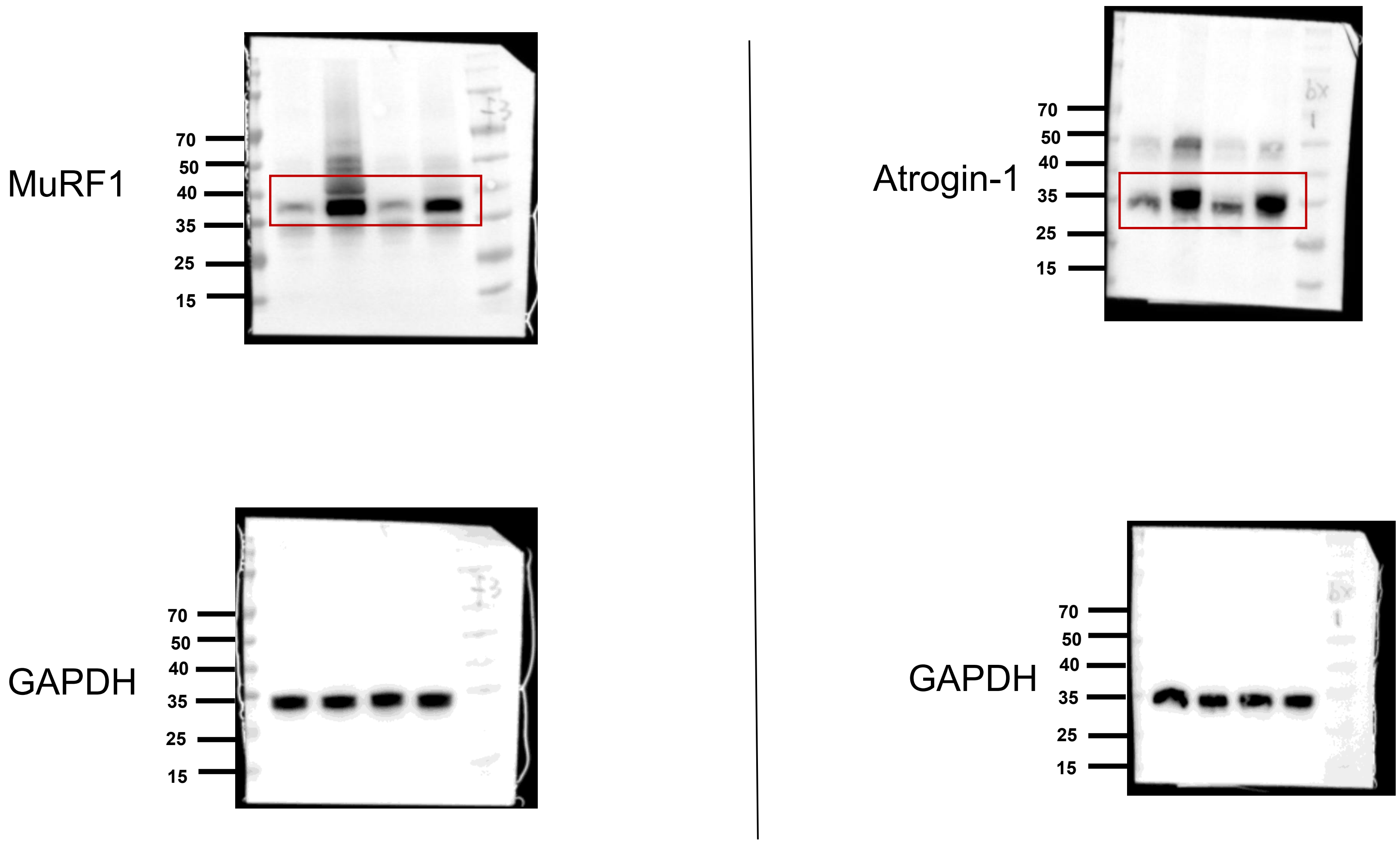

Supplemental Materials to Fig. 2D and 2F

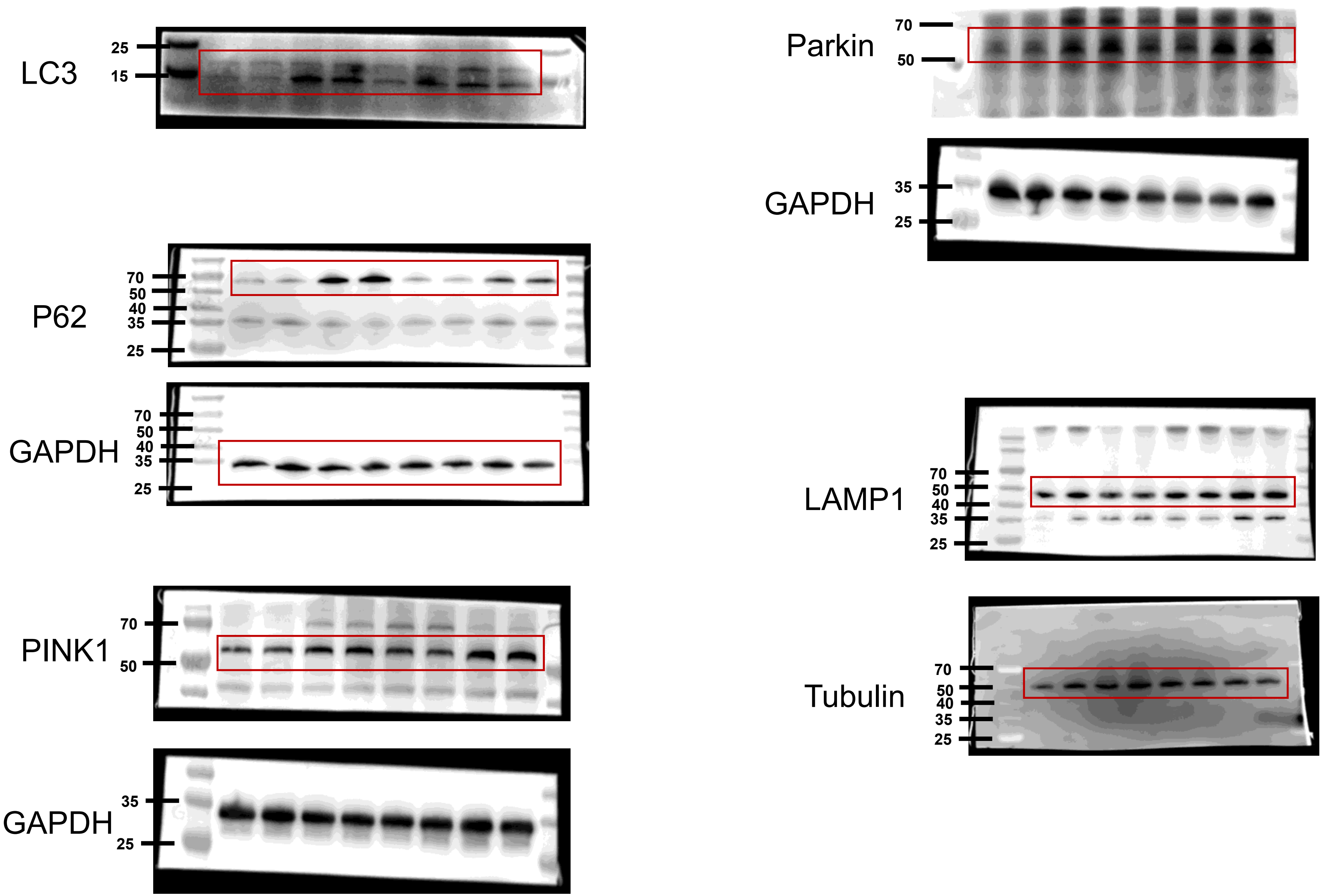

Supplemental Materials to Fig. 3B

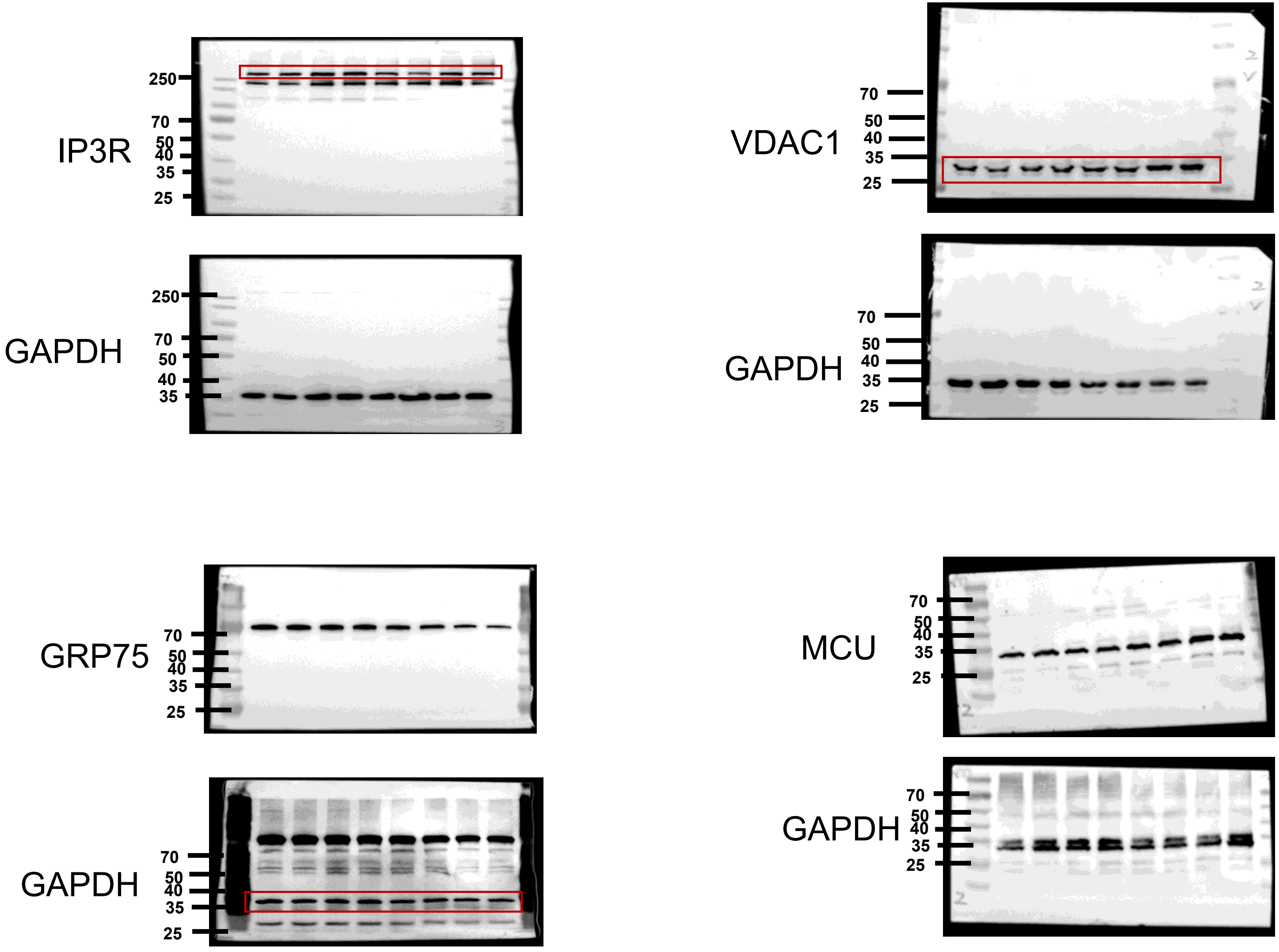

Supplemental Materials to Fig. 3I

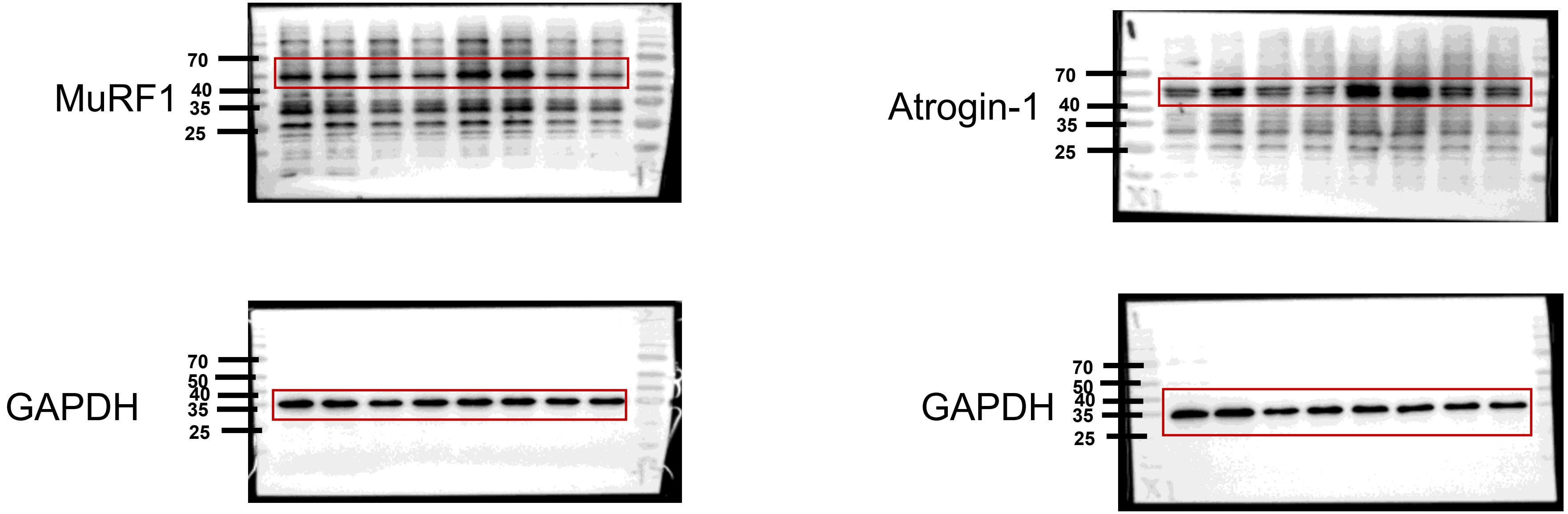

Supplemental Materials to Fig. 3M

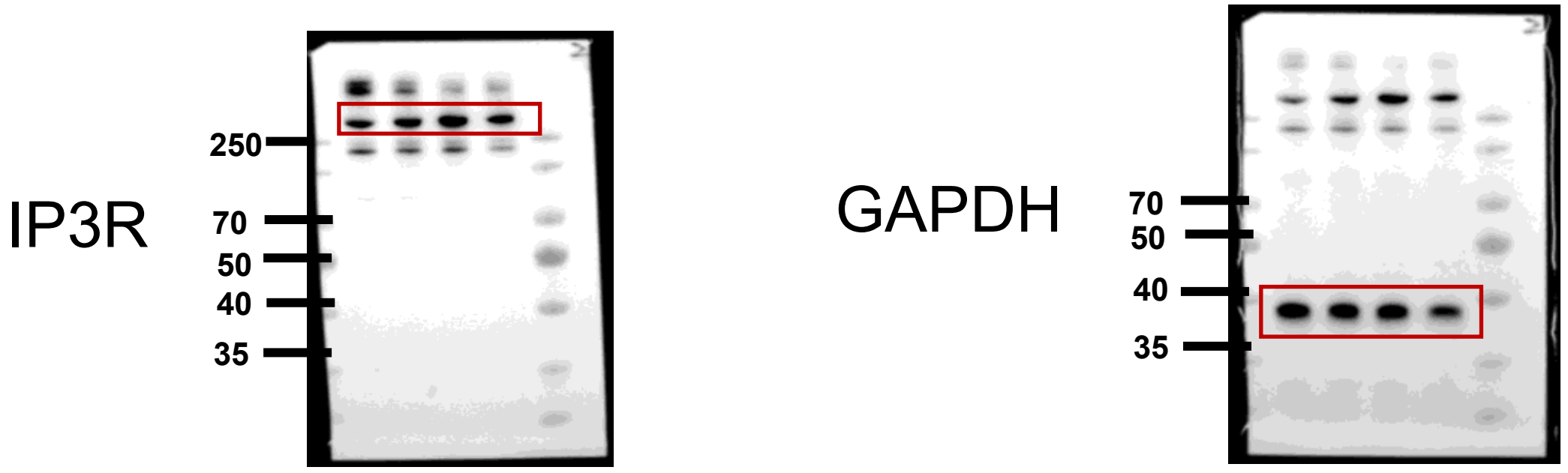

Supplemental Materials to Fig. 4C

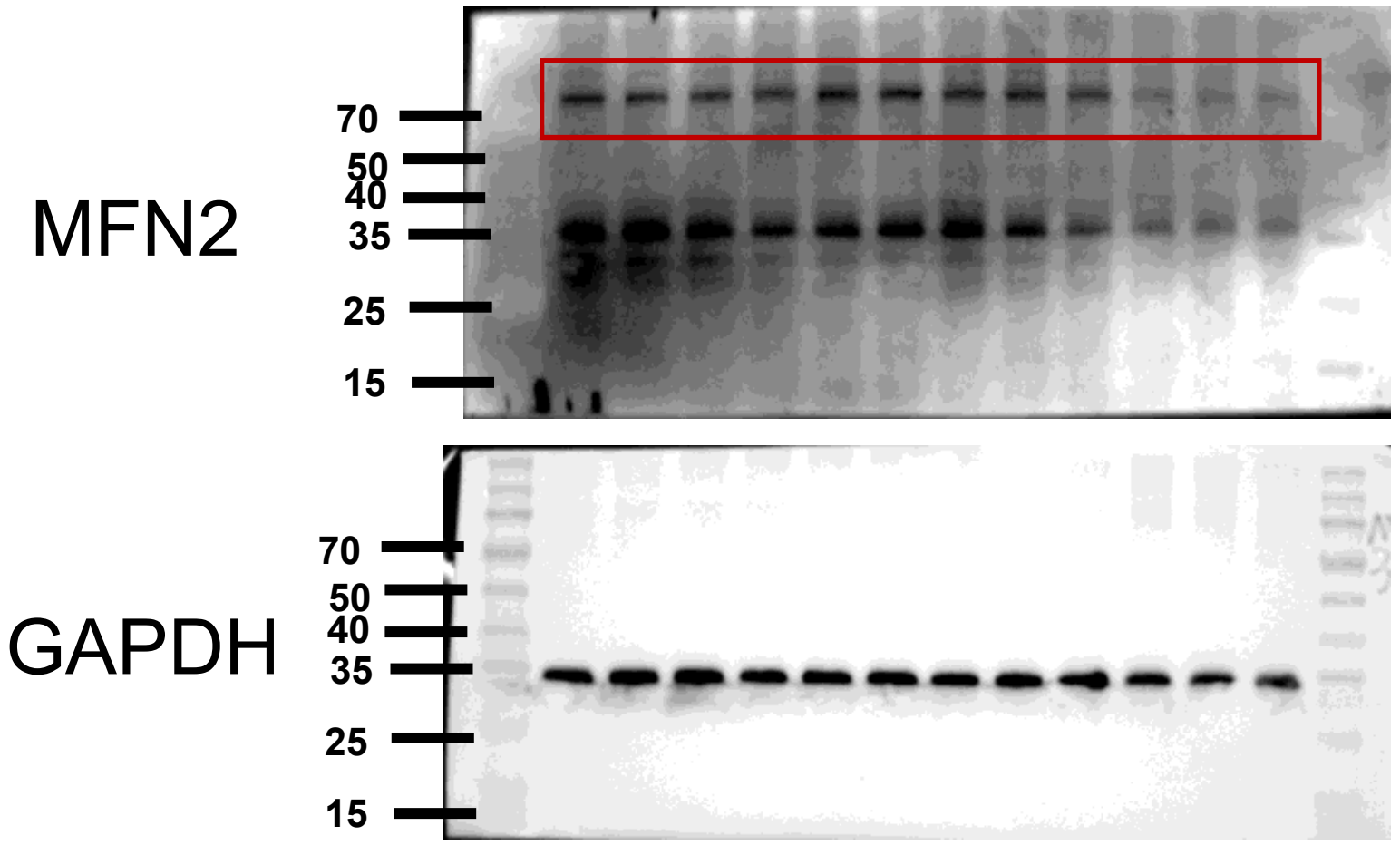

Supplemental Materials to Fig. 5A

MFN2 (TA)

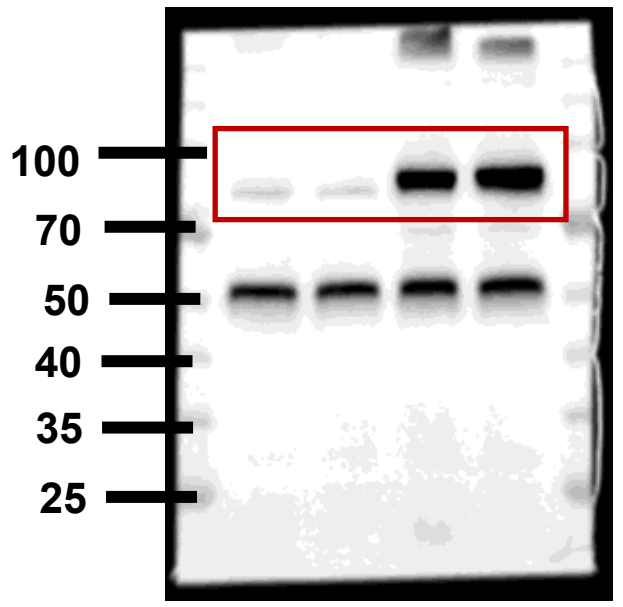

GAPDH

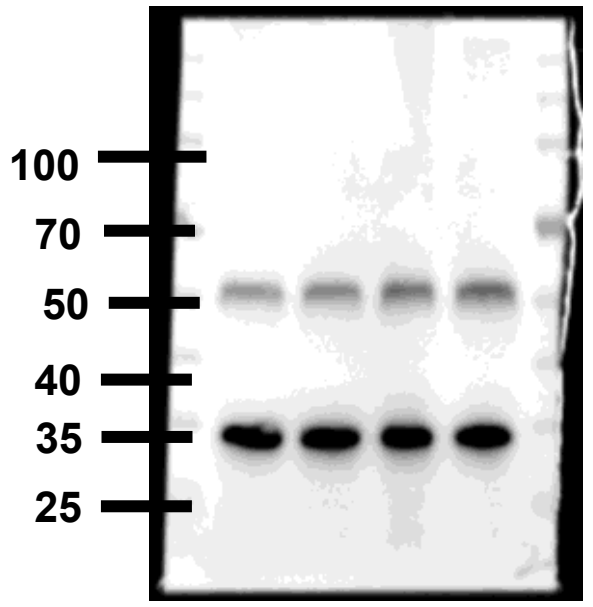

MFN2 (GAS)

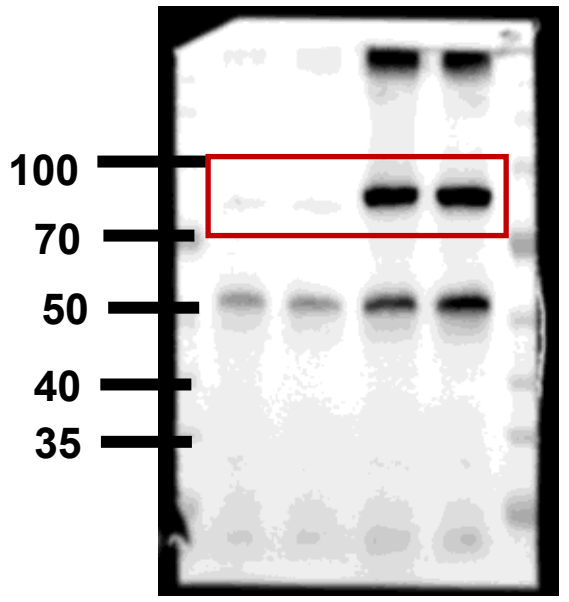

GAPDH

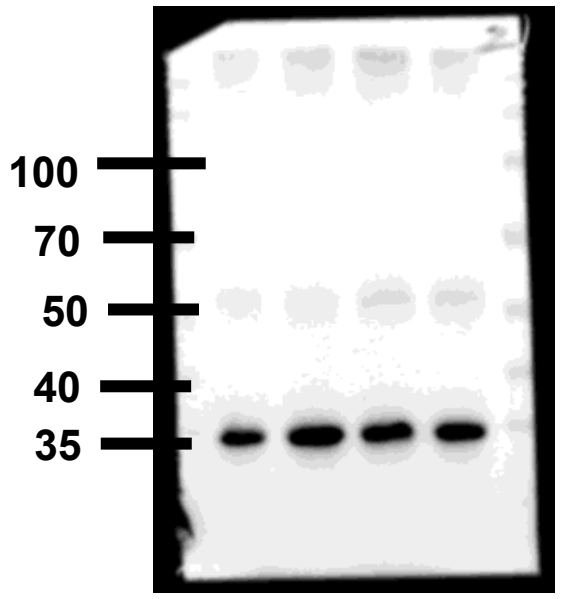

Supplemental Materials to Fig. 6G

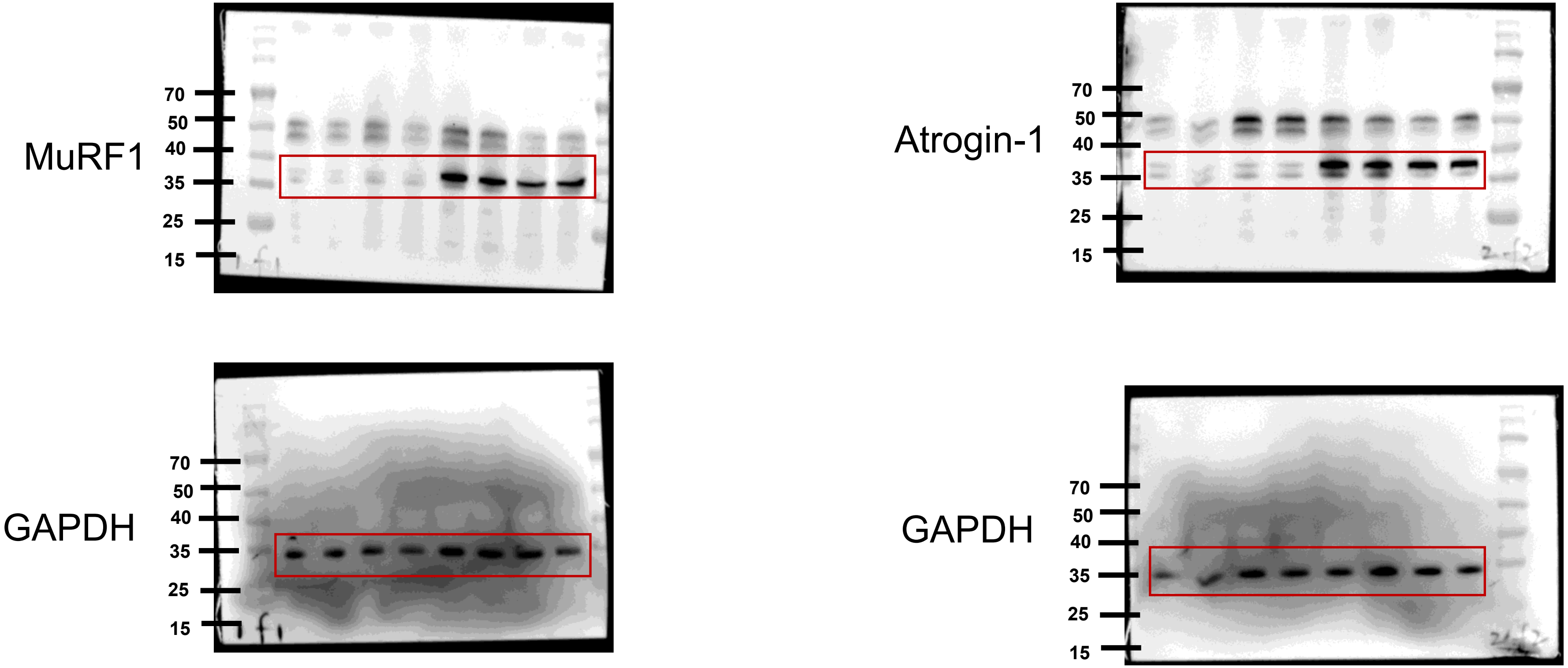

Supplemental Materials to Fig. 7C

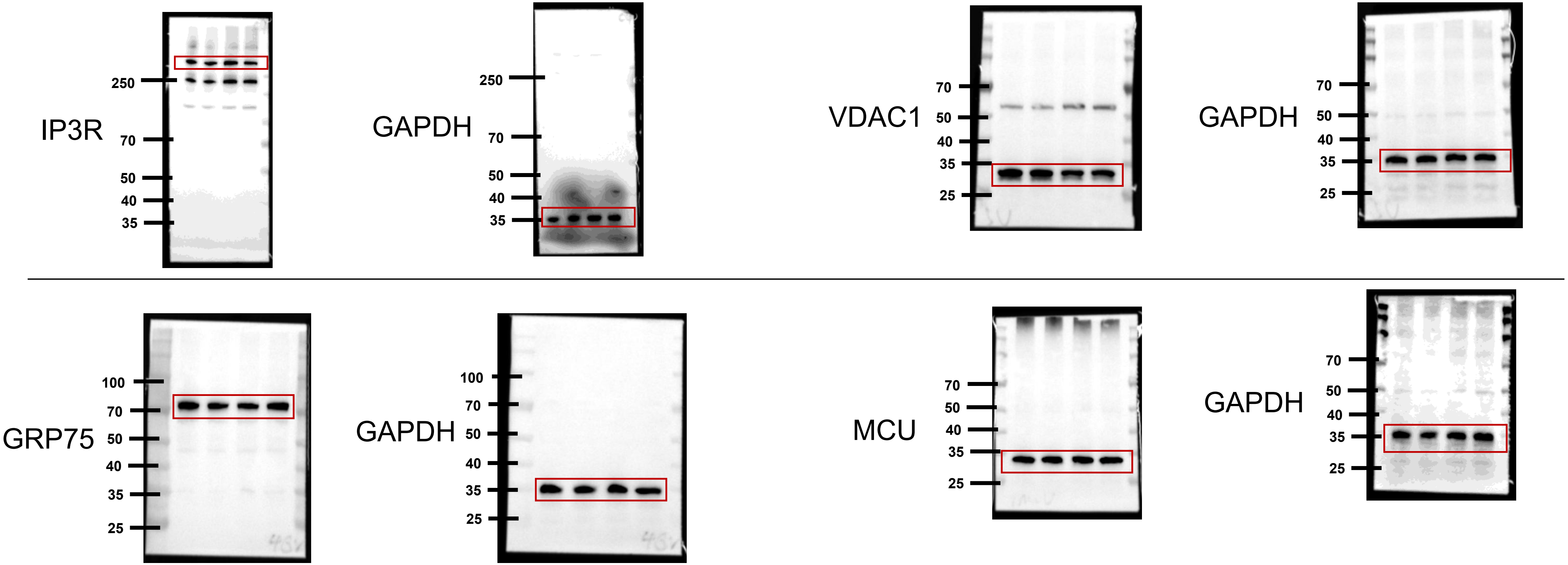

Supplemental Materials to Fig. 7E

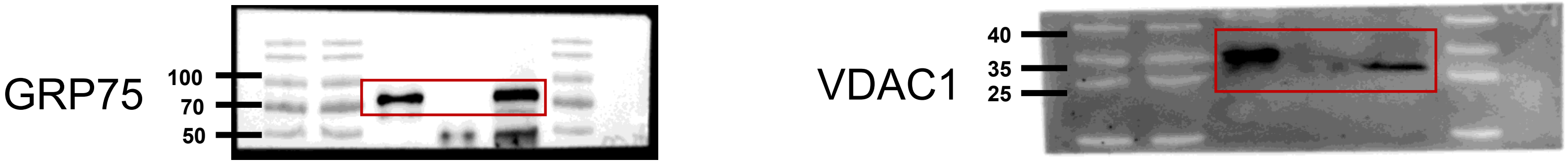

Supplemental Materials to Supplemental Fig. S2A

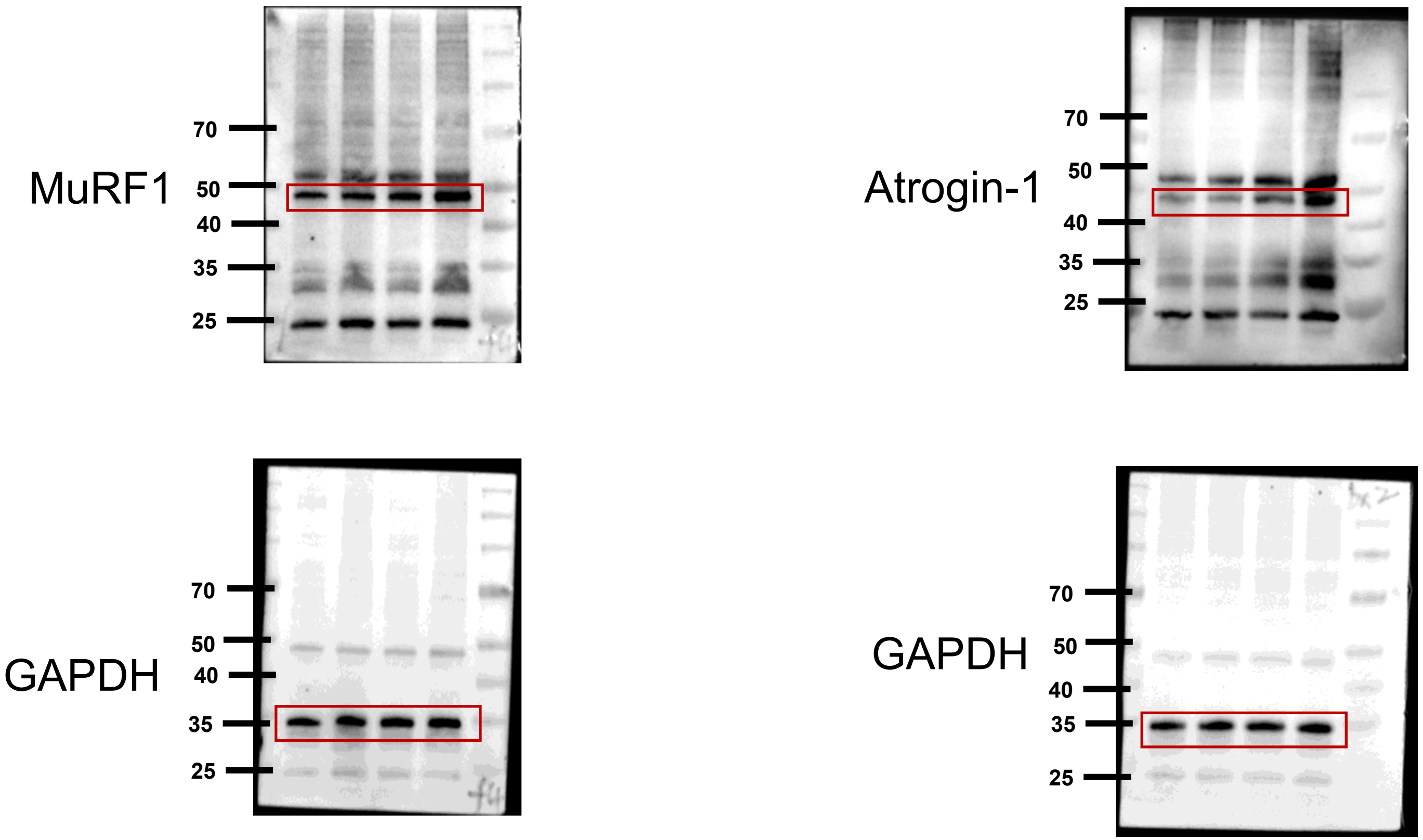

Supplemental Materials to Supplemental Fig. S3A

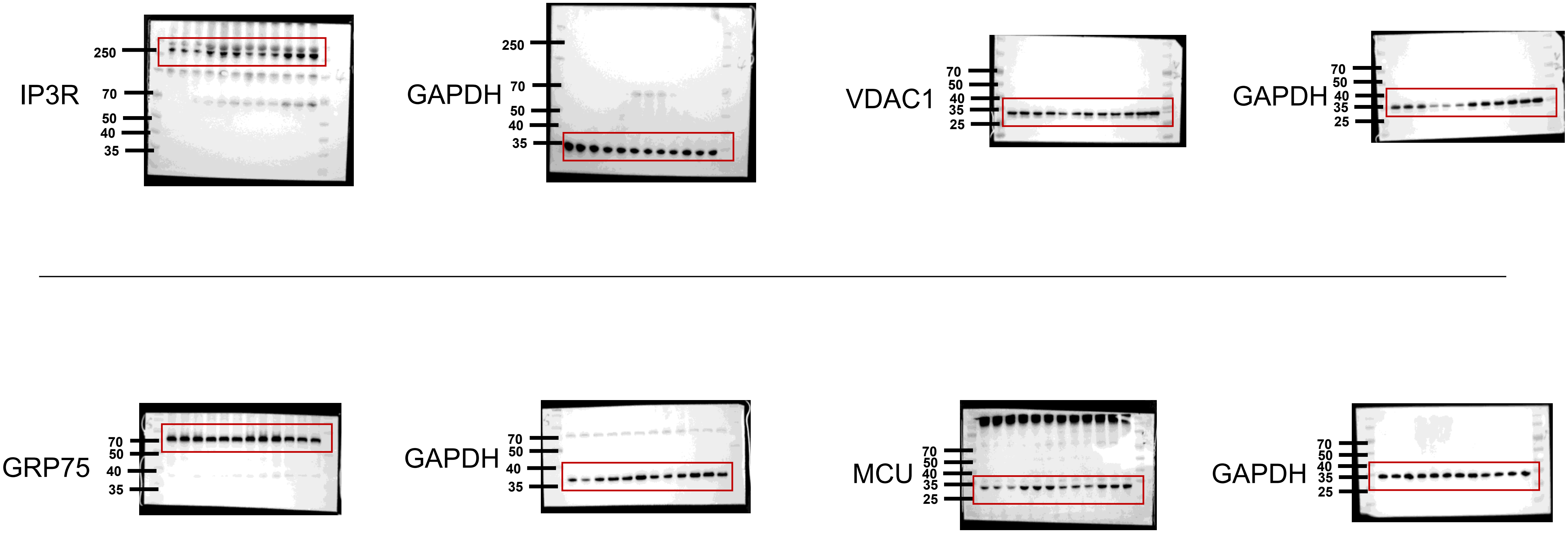

Supplemental Materials to Supplemental Fig. S4A

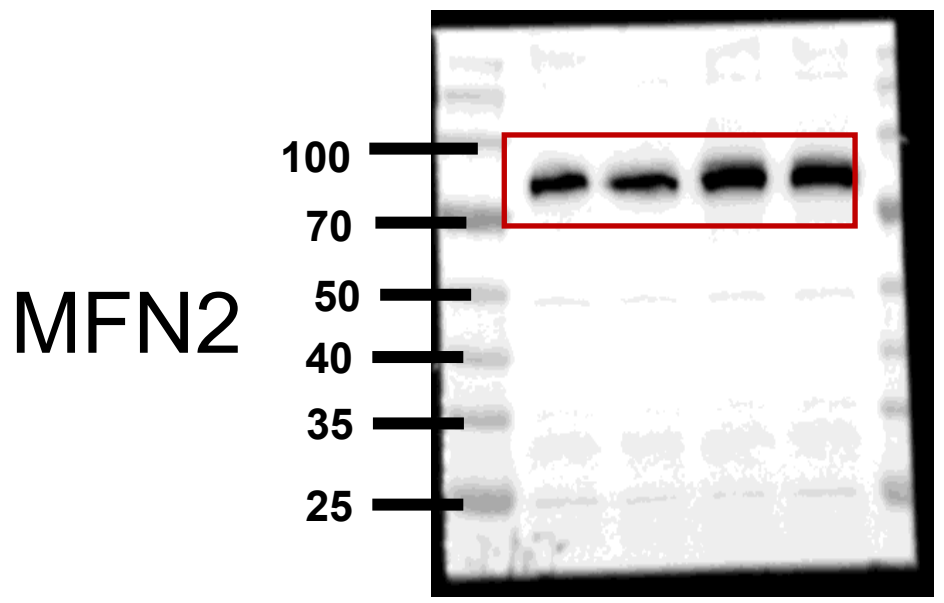

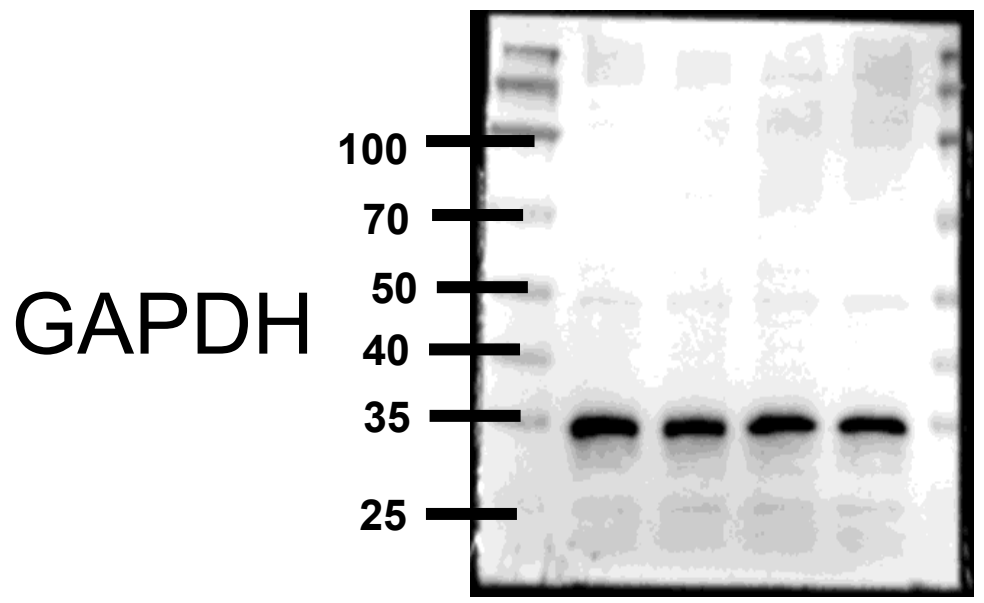

Supplemental Materials to Supplemental Fig. S4C

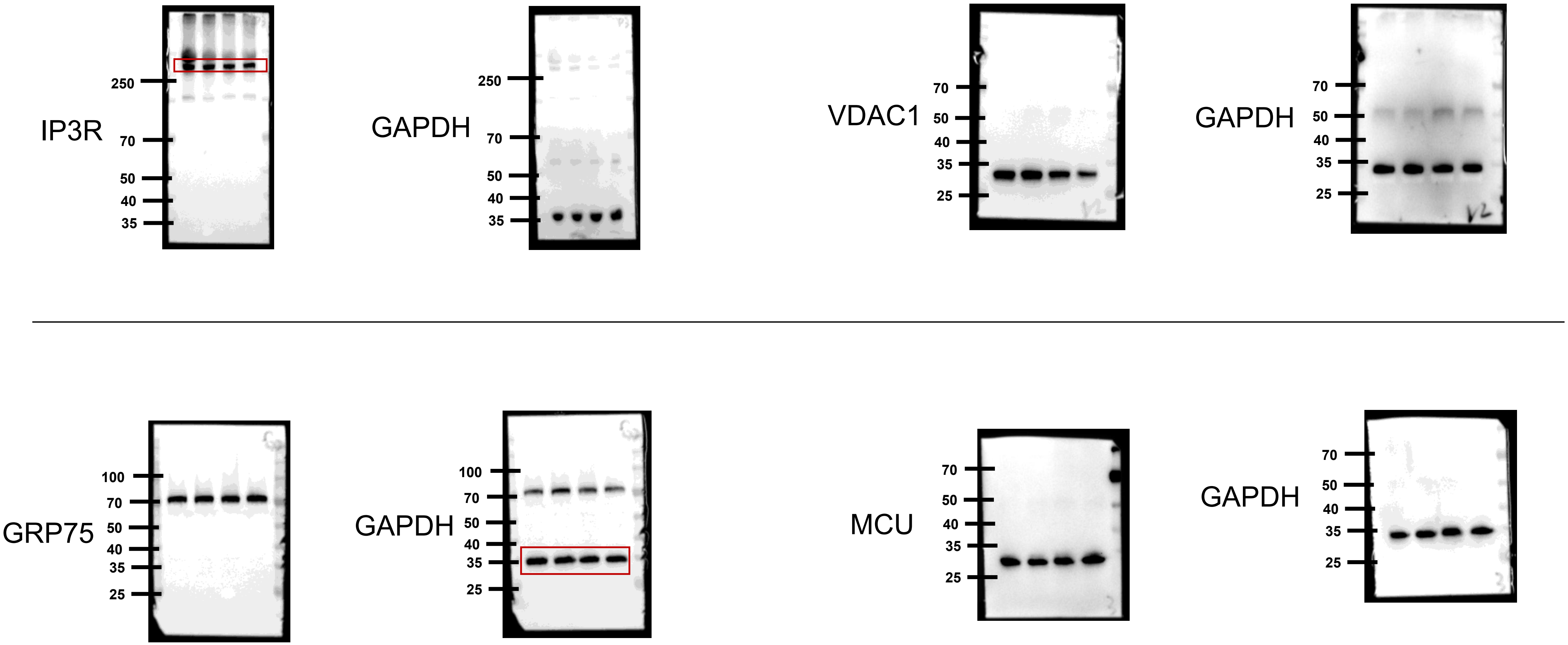

Supplement: Supplementary file 1 [file 13395_2026_441_MOESM2_ESM.pdf]
