# Supplementary material for: MFN2 alleviates mitochondrial calcium overload in sepsis-induced myopathy by modulating GRP75–VDAC1 interaction
Source: Skelet Muscle. 2026 Aug 13;16:41. doi: 10.1186/s13395-026-00441-6 (PMC13749854; doi:10.1186/s13395-026-00441-6)
Supplement: Supplementary file 2 [file 13395_2026_441_MOESM1_ESM.docx]

**Supplementary Figure 1**

**Supplementary Figure 1. Successful establishment of a sepsis model in mice.**

(A) Kaplan–Meier survival curves of sham and cecal ligation and puncture (CLP)-operated mice monitored at 24 h and 96 h post-surgery; *n* = 10 animals per group. (B) Changes in body weight of sham and CLP-operated mice following surgery; *n* = 5 animals per group. (C-E) Quantification of serum concentrations of the pro-inflammatory cytokines IL-6, TNF-α, and IL-1β at 24 h and 96 h following sham surgery or CLP; *n* = 4 animals per group. All data are presented as means ± SD. Survival curves in panel A were compared using the log-rank (Mantel-Cox) test. Statistical comparisons across the 2×2 factorial design (model: sham vs. CLP; time point: 24 h vs. 96 h) for panels B-E were performed using two-way ANOVA followed by Tukey's honestly significant difference (HSD) post hoc test. **P***＜**0.05; ***P***＜**0.01; ****P***＜**0.001; *****P***＜**0.0001.

**Supplementary Figure 2**

**Supplementary Figure 2. Establishment of an in vitro sepsis model in differentiated C2C12 myotubes.**

(A) Representative western blot images showing the protein expression of MuRF1 and Atrogin-1 in differentiated C2C12 myotubes treated with 2% (v/v) serum collected from sham-operated (SMS) or CLP-operated (CMS) mice, with (B) quantitative analysis of MuRF1 and Atrogin-1 protein levels normalized to GAPDH; *n* = 5 independent cell culture experiments. (C) Representative bright-field microscopy images of differentiated C2C12 myotubes following 6 days of differentiation induction, with (D) quantitative analysis of myotube diameters (30-40 myotubes measured per sample); *n* = 3 independent cell culture experiments. All data are presented as means ± SD. Statistical comparisons across the 2×2 factorial design (model: SMS vs. CMS; time point: 24 h vs. 96 h) were performed using two-way ANOVA followed by Tukey's HSD post hoc test. **P***＜**0.05; ***P***＜**0.01; ****P***＜**0.001; *****P***＜**0.0001.

**Supplementary Figure 3**

**Supplementary Figure 3. Alterations in IP3R–GRP75–VDAC1 Complex Protein Expression in the Tibialis Anterior Muscle of Septic Mice.**

(A) Representative western blot images showing the protein expression of IP3R, GRP75, VDAC1, and MCU in the tibialis anterior (TA) muscle of sham and CLP-operated mice at 24 h and 96 h post-surgery, with (B–E) quantitative analysis of each target protein normalized to GAPDH; *n* = 5 muscles per group. All data are presented as means ± SD. Statistical comparisons across the 2×2 factorial design (model: sham vs. CLP; time point: 24 h vs. 96 h) were performed using two-way ANOVA followed by Tukey's HSD post hoc test. **P***＜**0.05; ***P***＜**0.01; ****P***＜**0.001; *****P***＜**0.0001.

**Supplementary Figure 4**

**Supplementary Figure 4. Validation of MFN2 overexpression in C2C12 myoblasts and its effects on the IP3R–GRP75–VDAC1 complex expression in the TA muscles of septic mice.**

(A) Representative western blot images confirming MFN2 overexpression in C2C12 myoblasts following lentiviral vector (LV) transduction, with (B) quantitative analysis of MFN2 expression at both the protein levels (normalized to GAPDH) and mRNA levels; *n* = 5 independent cell culture experiments. (C) Representative western blot images showing the protein expression levels of IP3R, GRP75, VDAC1, and MCU in the TA muscles of mice following AAV-mediated MFN2 overexpression, with (D) quantitative analysis of each target protein normalized to GAPDH; *n* = 5 muscles per group. All data are presented as means ± SD. For panels C-D, contralateral muscles from the same animal injected with AAV-CON served as within-subject paired internal controls; *n* = 5 animals per group, with each animal contributing one AAV-MFN2 muscle and one contralateral AAV-CON muscle (paired design). For panels A-D, between-group comparisons were performed using unpaired two-tailed Student's t-tests. **P***＜**0.05; ***P***＜**0.01; ****P***＜**0.001; *****P***＜**0.0001.
