# Supplementary material for: MFN2 alleviates mitochondrial calcium overload in sepsis-induced myopathy by modulating GRP75–VDAC1 interaction
Source: Skelet Muscle. 2026 Aug 13;16:41. doi: 10.1186/s13395-026-00441-6 (PMC13749854; doi:10.1186/s13395-026-00441-6)
Supplement: Supplementary file 3 [file 13395_2026_441_MOESM3_ESM.docx]

**Supplementary Table 1 Antibody information for Western blot**

| Name of antibody | Dilution Ratio | Catalog Number | Manufacturers |
| --- | --- | --- | --- |
| MFN2 | 1:1000 | Sc-100560 | Senta Crue Biotechnology |
| IP3R | 1:1000 | Ab5804 | Abcam |
| GRP75 | 1:10000 | 14887-1-AP | proteintech |
| VDAC1 | 1:1000 | Ab14734 | Abcam |
| MCU | 1:1000 | 14997S | Cell Signaling Technology |
| MuRF1 | 1:1200 | Sc-398608 | Senta Crue Biotechnology |
| Atrogin-1 | 1:1200 | Sc-166806 | Senta Crue Biotechnology |
| LC3B | 1:1000 | ab192890 | Abcam |
| P62 | 1:5000 | ab109012 | Abcam |
| PINK1 | 1:1000 | 23274-1-AP | proteintech |
| Parkin | 1:1000 | 14060-1-AP | Proteintech |
| LAMP1 | 1:1000 | 21997-1-AP | proteintech |
| GAPDH | 1:1000 | sc-32233 | Senta Crue Biotechnology |
