# Supplementary material for: MFN2 alleviates mitochondrial calcium overload in sepsis-induced myopathy by modulating GRP75–VDAC1 interaction
Source: Skelet Muscle. 2026 Aug 13;16:41. doi: 10.1186/s13395-026-00441-6 (PMC13749854; doi:10.1186/s13395-026-00441-6)
Supplement: Supplementary file 4 [file 13395_2026_441_MOESM4_ESM.docx]

**Supplementary Table 2 Antibody information for immunofluorescence staining**

| Name of antibody | Dilution Ratio | Catalog Number | Manufacturers |
| --- | --- | --- | --- |
| IP3R | 1:200 | Ab5804 | Abcam |
| VDAC1 | 1:200 | Ab14734 | Abcam |
| GRP75 | 1:400 | 14887-1-AP | Proteintech |
| LAMP1 | 1:400 | 21997-1-AP | Proteintech |
